# Indigoidine and deoxyviolacein co-pigmentation in a single bacterial cell enables visual discrimination of Pb(ii) and Hg(ii) in environmental water matrices

**DOI:** 10.1039/d6ra05888d

**Published:** 2026-10-19

**Authors:** Zongjie Ma, Yan Guo, Wenjun Luo, Keyan Wei, Zhaobo Hu, Jiajun Huang, Yuting Chen, Chang-ye Hui

**Affiliations:** a Department of Toxicology, School of Public Health, Southern Medical University Guangzhou 510515 China; b Shenzhen Prevention and Treatment Center for Occupational Diseases Shenzhen 518020 China hcy_sypu@hotmail.com; c School of Public Health, Southern Medical University Guangzhou 510515 China; d School of Public Health, Shenzhen University Medical School, Shenzhen University Shenzhen 518055 China; e School of Public Health, Guangdong Medical University Dongguan 523808 China

## Abstract

Heavy metal co-contamination of lead and mercury in aquatic environments poses escalating risks to public health and ecosystems. Conventional instrumental methods, while accurate, are ill-suited for routine on-site screening due to high costs and operational complexity. Here, we describe a dual-pigment whole-cell biosensor engineered in *Escherichia coli* TOP10 for simultaneous visual detection of Pb(ii) and Hg(ii). The sensor integrates a Pb(ii)-responsive PbrR regulatory module that drives indigoidine biosynthesis with an Hg(ii)-responsive MerR module that drives deoxyviolacein biosynthesis within a single construct. Upon exposure to metal, the engineered strain produces distinct blue and purple pigments that are quantifiable at 600 nm and 570 nm, respectively. The biosensor achieved limits of detection of 6.74 nM for Pb(ii) and 0.0631 nM for Hg(ii), with both channels exhibiting high selectivity against competing metal ions. Performance was further validated in spiked surface water and seawater matrices, where clear dose–response relationships were maintained. By enabling naked-eye discrimination of two toxic metals near regulatory thresholds in a standard microplate format, this platform offers a low-cost, high-throughput alternative for preliminary environmental monitoring and emergency pollution assessment.

## Abstract 2

A single bacterial host engineered for indigoidine and deoxyviolacein co-pigmentation enables visual discrimination of Pb(ii) and Hg(ii) in environmental water matrices through intrinsic aqueous–organic phase separation.

## Graphical abstract

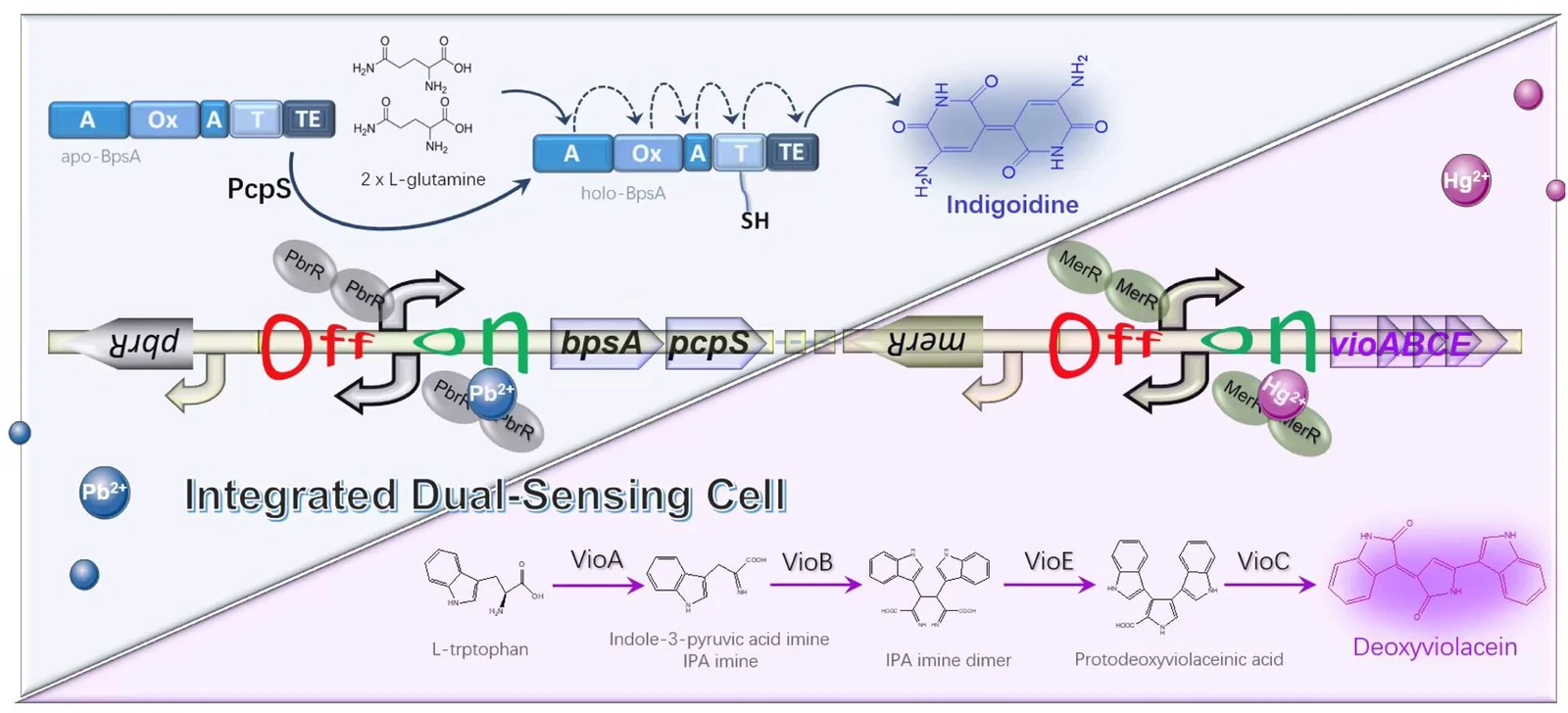

## Introduction

The heavy metals lead and mercury are notorious for their persistence in the environment, propensity for bioaccumulation, and deleterious impacts on human physiology and ecological integrity.^1,2^ Industrial operations spanning mining, smelting, battery production, and chemical manufacturing routinely release these pollutants into surface waters, creating matrices in which lead and mercury co-occur.^3^ Reports of such combined contamination are accumulating globally, and concurrent exposure to both metals amplifies toxicological risks to neurological, renal, and hematopoietic functions.^4,5^

Established analytical protocols for quantifying lead and mercury in environmental matrices typically employ atomic absorption spectrometry, atomic fluorescence spectrometry, cold vapor atomic fluorescence spectrometry, or inductively coupled plasma mass spectrometry.^6^ While these methods deliver outstanding sensitivity and accuracy, they require costly instrumentation, labor-intensive sample preparation, and specialized operator expertise.^7^ Consequently, their deployment for routine surveillance remains restricted in resource-constrained contexts, and frequent large-scale screening—particularly when simultaneous multi-metal analysis is needed—faces substantial logistical obstacles. Electrochemical sensors and colorimetric assays have been investigated as alternatives for dual-metal detection. However, these approaches frequently exhibit inadequate sensitivity or poor selectivity, compromising their reliability under authentic field conditions.^8,9^

Whole-cell biosensors based on engineered bacteria present a compelling alternative platform. Living cells can self-replicate, which drives the per-assay cost toward a minimum.^10^ Measurable signals are produced through straightforward cultivation without sophisticated instrumentation, substantially lowering the technical barrier to detection. Despite these advantages, the majority of reported bacterial biosensors have been configured for single-metal recognition.^11,12^ When several metals are screened in parallel, identical reporter outputs are typically deployed for each target, producing monochromatic signals that cannot be distinguished by eye.^13,14^ This configuration yields limited diagnostic information and precludes rapid discrimination of exposure levels or the relative contributions of individual pollutants in mixed samples.

Visual biosensors circumvent this constraint by permitting direct naked-eye readout at concentrations near regulatory thresholds. Reporter systems based on natural microbial pigments are especially well suited for this purpose. A growing palette of microbial pigments—including water-soluble blue indigoidine,^15^ purple deoxyviolacein,^16^ blue–purple violacein and its derivatives,^17^ orange anthocyanin,^18^ yellow carotenoid,^19^ black pyomelanin,^20^ blue indigo,^21^ and red prodigiosin^22^—has been recruited as chromogenic outputs. These distinct color signatures enable instrument-free deployment in low-resource settings. To date, however, research efforts have focused predominantly on single-metal sensors that emit a single chromogenic signal.^23,24^ The merger of multiple metal-responsive regulatory modules with distinct pigment biosynthetic pathways within a single cellular host has received little attention.

Although our group has previously reported a dual-color whole-cell biosensor that couples Pb(ii)-responsive prodeoxyviolacein (PDV) with Hg(ii)-responsive deoxyviolacein (DV) in a single host,^13^ that system relies on PDV—an unstable biosynthetic intermediate that requires chemical oxidation to develop a brown–green color and must be extracted into the same organic phase as DV, which limits signal stability and prevents physical separation of the two readouts. In the present study, we therefore replaced the PDV output with indigoidine (IND). This water-soluble and chemically stable blue pigment is quantified directly in the aqueous supernatant (*A*_600_), whereas DV remains quantified in the *n*-butanol extract (*A*_570_). This IND/DV pairing achieves three practical advances over the PDV/DV architecture: (i) elimination of the oxidation step and improved chemical stability; (ii) intrinsic aqueous/organic phase separation that minimizes mutual signal dilution and is experimentally validated (Fig. S4); and (iii) a sharper blue-*versus*-purple color contrast that is more readily distinguished by the naked eye.

Here, we report the construction and validation of this IND/DV dual-pigment biosensor. A Pb(ii)-responsive PbrR module drives IND biosynthesis while an Hg(ii)-responsive MerR module drives DV biosynthesis, both operating within a single *E. coli* TOP10 host. The resulting strain generates distinct blue and purple colors that enable simultaneous differential detection of both toxic metals near regulatory thresholds in a standard microplate format, offering a low-cost, high-throughput platform for preliminary environmental monitoring and emergency pollution assessment.

## Materials and methods

### Bacterial strains, plasmids, and reagents

*Escherichia coli* (*E. coli*) TOP10 harboring recombinant plasmids was routinely cultivated in Luria–Bertani (LB) broth (5 g L^−1^ yeast extract, 10 g L^−1^ tryptone, 10 g L^−1^ sodium chloride) supplemented with 50 µg mL^−1^ ampicillin. Cultivation of the seed culture was carried out at 37 °C with shaking at 250 rpm. Stock solutions of HgCl_2_, Pb(NO_3_)_2_, CdCl_2_, ZnSO_4_, MgSO_4_, CuSO_4_, MnSO_4_, and CaCl_2_ were freshly prepared using analytical-grade reagents (Aladdin, Shanghai, China). Oligonucleotide primers and synthetic DNA fragments were obtained from Sangon Biotech (Shanghai, China).

### Plasmid construction and biosensor assembly

The dual-target sensing plasmid was constructed through two sequential cloning steps. First, the Pb(ii) sensing module was assembled using an uncoupled genetic circuit that incorporates the PbrR regulator and its divergent promoter P*pbr* derived from the *Klebsiella pneumoniae* CG43 plasmid pLVPK.^25^ The output module comprised the indigoidine (IND) biosynthetic gene cluster *bpsA-pcpS*.^26^ Second, the Hg(ii) sensing module was assembled using an uncoupled circuit that harbors the MerR regulator and its divergent promoter P*mer* from *E. coli*.^27^ The output module comprised the deoxyviolacein (DV) biosynthetic gene cluster *vioABCE*.^28^ These two modules were inserted into the pET-21a backbone at the *Bgl*II–*Sac*I and *Sac*I–*Hind*III sites, respectively, yielding the plasmid designated pPb-IND-Hg-DV. The resulting plasmid was transformed into *E. coli* TOP10 to generate the dual-target whole-cell biosensor. The detailed DNA sequence of the inserted fragment is provided in Table S1.

### Dose–response assays

#### Time–dose–response kinetics of indigoidine biosynthesis

To optimize the uniform induction conditions for simultaneous dual-pigment production, the time-course kinetics of IND biosynthesis were first characterized. An overnight seed culture of *E. coli* TOP10/pPb-IND-Hg-DV was grown in LB broth containing 50 µg mL^−1^ ampicillin at 37 °C with shaking at 250 rpm for 12 h, then diluted 1 : 100 (v/v) into fresh LB medium. Pb(ii) was added at 0, 25, and 50 µM final concentrations. From 4 to 11 h post-inoculation, 1 mL samples were collected at 1 h intervals.

For each sample, the optical density at 600 nm (OD_600_) was measured before centrifugation to record the total attenuance of the culture. The sample was then centrifuged at 12 000×*g* for 5 min, and the absorbance of the resulting supernatant at 600 nm (*A*_600_) was measured to quantify the water-soluble IND pigment. The corrected bacterial density, reflecting the true light-scattering contribution of the cells, was obtained by subtracting the supernatant *A*_600_ from the whole-culture OD_600_.

This supernatant–subtraction approach follows standard practice for indigoidine-producing systems, in which IND remains in the aqueous phase while cells are pelleted.^26,29^ All absorbance readings were performed using a microplate reader (BioTek Epoch, Winooski, VT, USA).

#### Pb(ii) dose–response assay

A single colony of *E. coli* TOP10/pPb-IND-Hg-DV was inoculated into LB broth containing 50 µg mL^−1^ ampicillin and incubated overnight at 37 °C with shaking at 250 rpm to prepare the seed culture. The next day, the culture was diluted 1 : 100 (v/v) into 1 mL fresh LB medium supplemented with 50 µg mL^−1^ ampicillin in a 15 mL centrifuge tube. A two-fold serial dilution of Pb(ii) standard solution was added to establish final concentrations of 0, 0.003, 0.006, 0.012, 0.024, 0.049, 0.098, 0.195, 0.391, 0.781, 1.563, 3.125, 6.25, 12.5, 25, 50, 100, and 200 µM. All treatment groups were incubated at 30 °C with shaking at 150 rpm for 10 h. Bacterial density was measured at OD_600_. The culture supernatant was collected after centrifugation, and the absorbance of IND was determined at 600 nm using a microplate reader.

#### Hg(ii) dose–response assay

A single colony was inoculated into LB broth containing 50 µg mL^−1^ ampicillin and incubated overnight at 37 °C with shaking at 250 rpm. The following day, the culture was diluted 1 : 100 (v/v) into 1 mL fresh LB medium containing 50 µg mL^−1^ ampicillin in a 15 mL centrifuge tube. A two-fold serial dilution of Hg(ii) standard solution was added to achieve final concentrations of 0, 0.153, 0.305, 0.61, 1.221, 2.441, 4.883, 9.766, 19.531, 39.063, 78.125, 156.25, 312.5, 625, 1250, 2500, and 5000 nM. All groups were incubated at 30 °C with shaking at 150 rpm for 10 h. Bacterial density was monitored at OD_600_. DV was extracted with *n*-butanol, and the absorbance was measured at 570 nm.^30^

### Phase-partitioning validation of pigment distribution

IND and DV were prepared independently, and their distribution between the aqueous and *n*-butanol phases was determined under the same conditions used for biosensor readout. Each pigment was produced by its cognate single-module strain under large-scale induction: IND was obtained as the culture supernatant of the IND-producing strain (harboring pPpbr-ind^15^ containing a *bpsA-pcpS* gene cluster), and DV as the *n*-butanol extract of the DV-producing strain (harboring pCon-DV^27^ containing a *vioABCE* gene cluster). An aliquot of each pigment preparation was combined with the complementary phase, vortexed thoroughly, and centrifuged at 12 000×*g* for 5 min to achieve complete phase separation. Following separation, 200 µL of the aqueous phase and 100 µL of the *n*-butanol phase were transferred to a microplate, and the absorbance of both phases was recorded at 570 nm and 600 nm (BioTek Epoch, Winooski, VT, USA). The distribution of each pigment was expressed as the volume-weighted percentage of the total absorbance recovered in each phase. The apparent partition coefficient was calculated as *K*_d_ = Absorbance (*n*-butanol phase)/Absorbance (aqueous phase), which quantifies the extent to which a pigment prefers the *n*-butanol phase. The correction factors, on the other hand, are used to calculate the true absorbance values of our pigment products at the corresponding wavelengths. The correction factor for removing the DV contribution from the IND channel is calculated as the absorbance of DV in the aqueous phase at 600 nm divided by the absorbance of DV in the *n*-butanol phase at 570 nm. The correction factor for removing the IND contribution from the DV channel is calculated as the absorbance of IND in the *n*-butanol phase at 570 nm divided by that in the aqueous phase at 600 nm.

### Metal selectivity assessment

An overnight culture of *E. coli* TOP10/pPb-IND-Hg-DV was prepared in LB broth containing 50 µg mL^−1^ ampicillin at 37 °C with shaking at 250 rpm. The next day, the culture was diluted 1 : 100 (v/v) into 2 mL fresh medium. Individual metal ions were added for exposure treatment. Given the high cytotoxicity of Hg(ii), three concentrations were set at 25, 50, and 100 nM. Pb(ii) and the remaining ions, including Mg(ii), Ca(ii), Cd(ii), Mn(ii), Cu(ii), and Zn(ii), exhibit lower cytotoxicity and were added at final concentrations of 2.5, 5, and 10 µM. All treatment groups were incubated at 30 °C with shaking at 150 rpm for 10 h. A blank control without metal addition was prepared concurrently.

After exposure, bacterial density was measured at OD_600_, and dual pigment signals were extracted and quantified. For IND detection, 900 µL of culture was centrifuged at 12 000×*g* for 5 min, and 200 µL of the resulting supernatant was transferred to a 96-well plate for absorbance measurement at 600 nm. For DV detection, 360 µL of *n*-butanol was added to the remaining culture. The mixture was vortexed thoroughly and centrifuged at 12 000×*g* for 5 min, and 100 µL of the upper organic phase was collected for absorbance measurement at 570 nm.

### Anti-interference assessment

#### Anti-interference evaluation of Pb(ii) response

An overnight seed culture was prepared and diluted 1 : 100 (v/v) into 2 mL fresh medium containing 50 µg mL^−1^ ampicillin. Binary metal co-exposure experiments were performed by adding Pb(ii) at a fixed final concentration of 5 µM to all treatment groups. Individual interfering metal ions, including Cd(ii), Zn(ii), Mg(ii), Cu(ii), Mn(ii), and Ca(ii), were spiked in separately at 5 µM each. The mixed-metal exposure systems were incubated at 30 °C with shaking at 150 rpm for 10 h. A positive control containing only 5 µM Pb(ii) and a negative control without metal addition were included in parallel. After exposure, bacterial density and dual-pigment signals were measured.

#### Anti-interference evaluation of Hg(ii) response

An overnight culture was diluted 1 : 100 (v/v) into fresh medium containing 50 µg mL^−1^ ampicillin. Binary co-exposure experiments were conducted by adding Hg(ii) at a fixed final concentration of 20 nM to all groups. Individual interfering metal ions, including Pb(ii), Cd(ii), Zn(ii), Mg(ii), Cu(ii), Mn(ii), and Ca(ii), were added separately at 5 µM each. The mixed exposure systems were incubated at 30 °C with shaking at 150 rpm for 10 h. A positive control with 20 nM Hg(ii) alone and a negative control without metal addition were prepared concurrently. After exposure, bacterial density and dual-pigment signals were analyzed.

### Determination of a two-dimensional cross-concentration matrix for interference assessment

To evaluate the independence of signals between the two detection channels and the extent of mutual interference under co-exposure conditions, a two-dimensional cross-concentration matrix experiment was conducted. *Escherichia coli* TOP10/pPb-IND-Hg-DV was cultured according to the standard operating procedures described above. A complete cross-matrix comprising 3 × 3 concentration combinations (including blank controls) was established. Hg(ii) was added at final concentrations of 0, 10, and 50 nM, while Pb(ii) was spiked at final concentrations of 0, 5, and 20 µM.

Cultivation was performed under uniform conditions for 10 hours. After incubation, the pigment signal was extracted according to the previously described method. Using the obtained absorbance data (*A*_570_ and *A*_600_), the crosstalk rate for each target metal was calculated. This crosstalk rate is defined as the relative percentage change in the target channel's absorbance caused by the non-target metal at a fixed concentration of the target metal.

### Performance in environmental water matrices

Surface water was collected from Honghu Park in Luohu District, Shenzhen, Guangdong Province, China. Seawater was collected from Dayawan Bay in Yantian District, Shenzhen, Guangdong Province, China. The samples were clarified by centrifugation at 10 000 rpm for 10 min and filtered through 0.22 µm membranes (Millipore, Billerica, MA, USA) to remove particulate matter and indigenous microorganisms. Atomic fluorescence spectrometry confirmed that the native concentrations of target metals in these samples were below the instrumental detection limits. Spiked samples were subsequently prepared for recovery experiments.

The detection culture systems were prepared by mixing environmental water samples, 10× LB, 10× NaCl-free LB, and sterile water according to previously reported proportions.^31^ Overnight cultures of TOP10/pPb-IND-Hg-DV were inoculated at 1% (v/v) into these detection systems containing 50 µg mL^−1^ ampicillin.

### Pb(ii) response in environmental water matrices

A two-fold serial dilution method was used to prepare Pb(ii) exposure gradients at final concentrations of 0, 0.003, 0.006, 0.012, 0.024, 0.049, 0.098, 0.195, 0.391, 0.781, 1.563, 3.125, 6.25, 12.5, 25, 50, 100, and 200 µM. The cultures were incubated at 30 °C with shaking at 150 rpm for 10 h. Bacterial density and IND pigment signals were measured as described above.

### Hg(ii) response in environmental water matrices

A two-fold serial dilution of Hg(ii) was added to achieve final concentrations of 0, 0.153, 0.305, 0.61, 1.221, 2.441, 4.883, 9.766, 19.531, 39.063, 78.125, 156.25, 312.5, 625, 1250, 2500, and 5000 nM. The cultures were incubated at 30 °C with shaking at 150 rpm for 10 h. Bacterial density and DV pigment signals were determined as described above.

### Statistical analysis

All experiments included at least three biological replicates. Data analysis was performed using IBM SPSS Statistics 26, and graphical plotting was completed using OriginPro 2024. Results are expressed as mean ± standard deviation (SD, *n* ≥ 3). When the data did not meet the assumptions of normality or homogeneity of variance, an appropriate transformation was applied prior to statistical testing. Comparisons between two groups were analyzed using Student's *t*-test. Comparisons among three or more groups were analyzed using one-way analysis of variance (ANOVA). A *P*-value less than 0.05 was considered statistically significant.

Full-range dose–response curves were fitted with a second-order polynomial model to describe the overall quantitative working range, which encompasses both the ascending induction phase and the subsequent decline caused by metal cytotoxicity at elevated concentrations; only the ascending (quasi-linear) portion was employed for quantitative determination, whereas the saturation plateau and cytotoxicity-induced decline were excluded from quantitative analysis. The limit of detection (LOD) was calculated according to the IUPAC criterion as LOD = 3*σ*/*k*, where *σ* is the standard deviation of the blank-sample responses and *k* is the slope of the linear calibration curve derived from the low-concentration linear segment of the dose–response data.^32^ Linear calibration for LOD determination was performed on the low-concentration ascending segments preceding signal saturation, specifically 0–1.221 nM for the Hg(ii)–DV channel and 0–0.024 µM for the Pb(ii)–IND channel; the blank standard deviation (*σ*) was computed from 3 independent blank replicates.

## Results and discussion

### Dual quantitative biosensing of Hg(ii) *via* DV and Pb(ii) *via* IND

The operating principle of the dual-target biosensor is illustrated in Fig. 1. In the absence of Pb(ii), the PbrR dimer binds to the divergent promoter P*pbr* and represses transcription of the downstream IND biosynthetic genes *bpsA* and *pcpS*. In the absence of Hg(ii), the MerR dimer binds to the divergent promoter P*mer* and blocks transcription of the downstream DV biosynthetic genes *vioABCE*. Upon entry of extracellular Pb(ii) or Hg(ii) into the cell, the metal ions bind to their respective dimeric regulators, triggering conformational changes that initiate expression of the downstream reporter genes. The resulting synthases catalyze the conversion of endogenous substrates into the corresponding pigments, IND and DV.^15,33^ Within a certain range of metal exposure concentrations, pigment yield shows a positive correlation with the concentration of the target metal ion, enabling simultaneous visual quantitative detection of both Pb(ii) and Hg(ii). Fig. S1 shows the full-wavelength absorption spectra of the color-forming products of Pb-indigoidine and Hg-DV. The characteristic absorption peak of DV is located near 570 nm, while that of indigoidine is located near 600 nm; these wavelengths are used to read the corresponding dye signals.

**Fig. 1:**
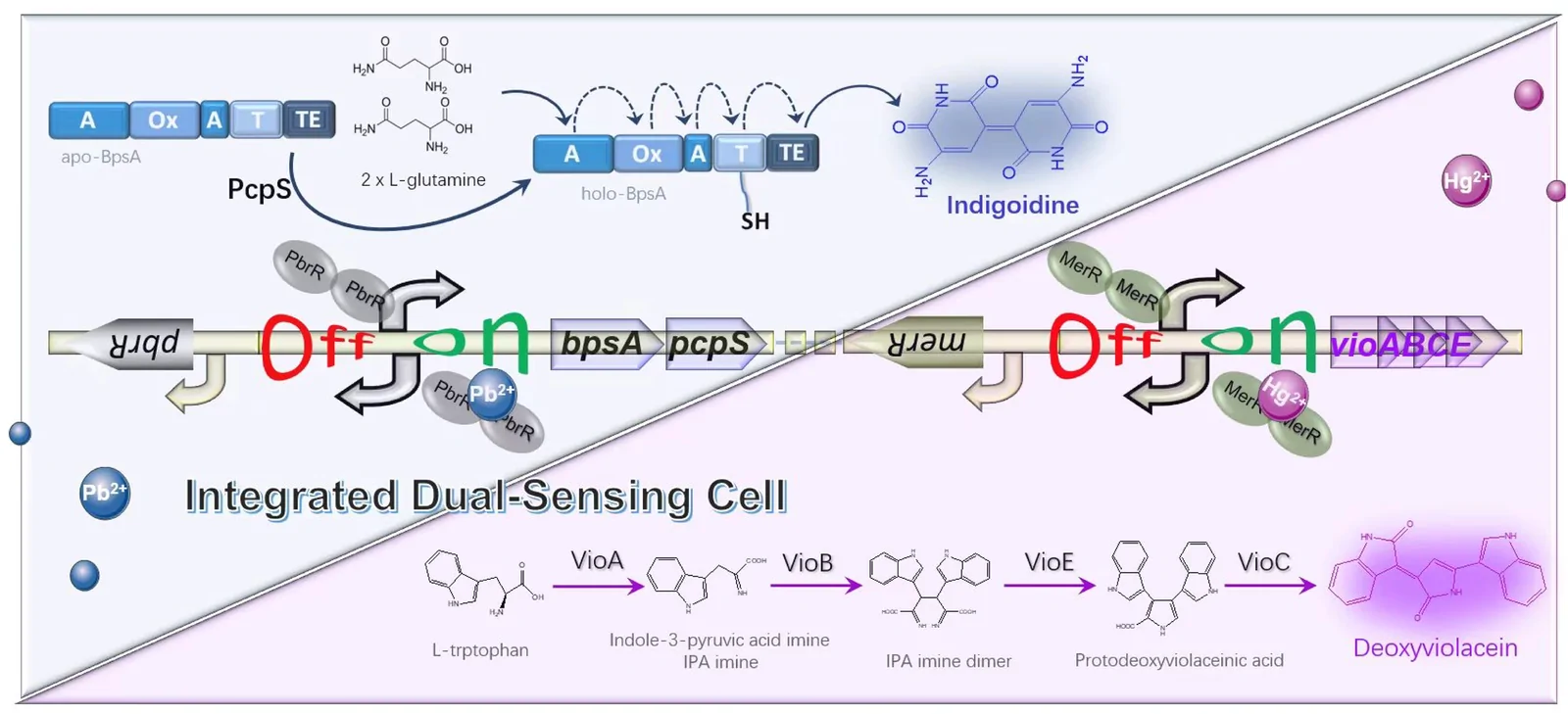
Schematic illustration of the dual-target whole-cell biosensor for Pb(ii) and Hg(ii) detection. In the absence of target metals, dimeric PbrR and MerR bind to their respective divergent promoters (P*pbr* and P*mer*) and repress downstream transcription. Upon metal entry, Pb(ii)-bound PbrR or Hg(ii)-bound MerR undergoes conformational activation, driving expression of the IND biosynthetic genes (*bpsA-pcpS*) or the DV biosynthetic genes (*vioABCE*). The resulting pigments enable differential colorimetric detection.

Fig. 2 shows the time–dose–response curves for Pb(ii)-induced indigoidine signal generation. The high concentrations of Pb(ii) used in this study did not exhibit significant cytotoxicity toward the sensor cells, as evidenced by the gradual increase in bacterial density over the culture period across all three Pb(ii) exposure concentrations, with no significant differences among the groups (Fig. 2A). As assessed by colorimetric quantification (Fig. 2B) and visual inspection (Fig. 2C), the increase in the indigoidine signal over time exhibited a clear dose–response relationship. At 10 hours, the intensity of the indigoidine signal produced by different Pb(ii) concentrations stabilized, and no further pigment was generated as time progressed. Therefore, 10 hours was selected as the exposure incubation time to develop the indigoidine signal.

**Fig. 2:**
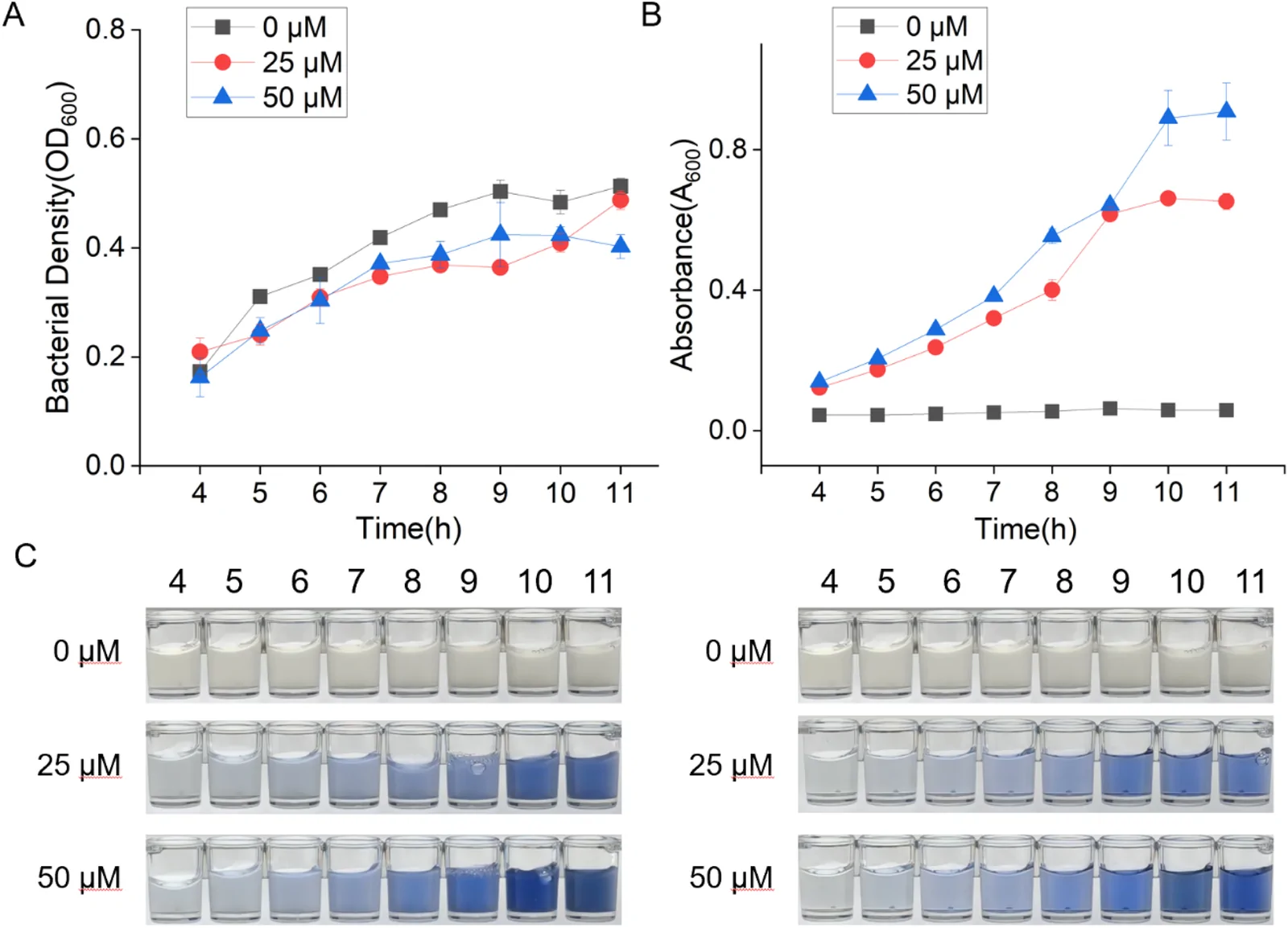
Time–dose–response curves for Pb(ii)-induced indigoidine signal generation. (A) Bacterial density (OD_600_) in the three Pb(ii) exposure groups at different time points. (B) Intensity of the Pb(ii)-induced indigoidine signal in the three Pb(ii) exposure groups at different time points. (C) Corresponding images of the colorimetric wells.

The engineered TOP10/pPb-IND-Hg-DV dual-target whole-cell biosensor exhibited favorable dose–response relationships toward both Pb(ii) and Hg(ii). For Hg(ii), the absorbance response at 570 nm increased with metal concentration. It reached a peak at 78.125 nM, followed by a decline at higher concentrations (Fig. 3B). For Pb(ii), the absorbance response at 600 nm showed a similar trend, peaking at 50 µM (Fig. 4B). This parabolic pattern is consistent with the cytotoxic effects of heavy metals on host cell viability. Once the metal concentration exceeds the engineered bacteria's tolerance threshold, growth inhibition occurs (Fig. 3A and 4A), thereby reducing reporter gene expression and, consequently, lowering pigment accumulation. We can observe changes in pigment color in the colorimetric cuvettes with the naked eye (Fig. 3 and 4), which is one of the most fascinating advantages of biosensors.

**Fig. 3:**
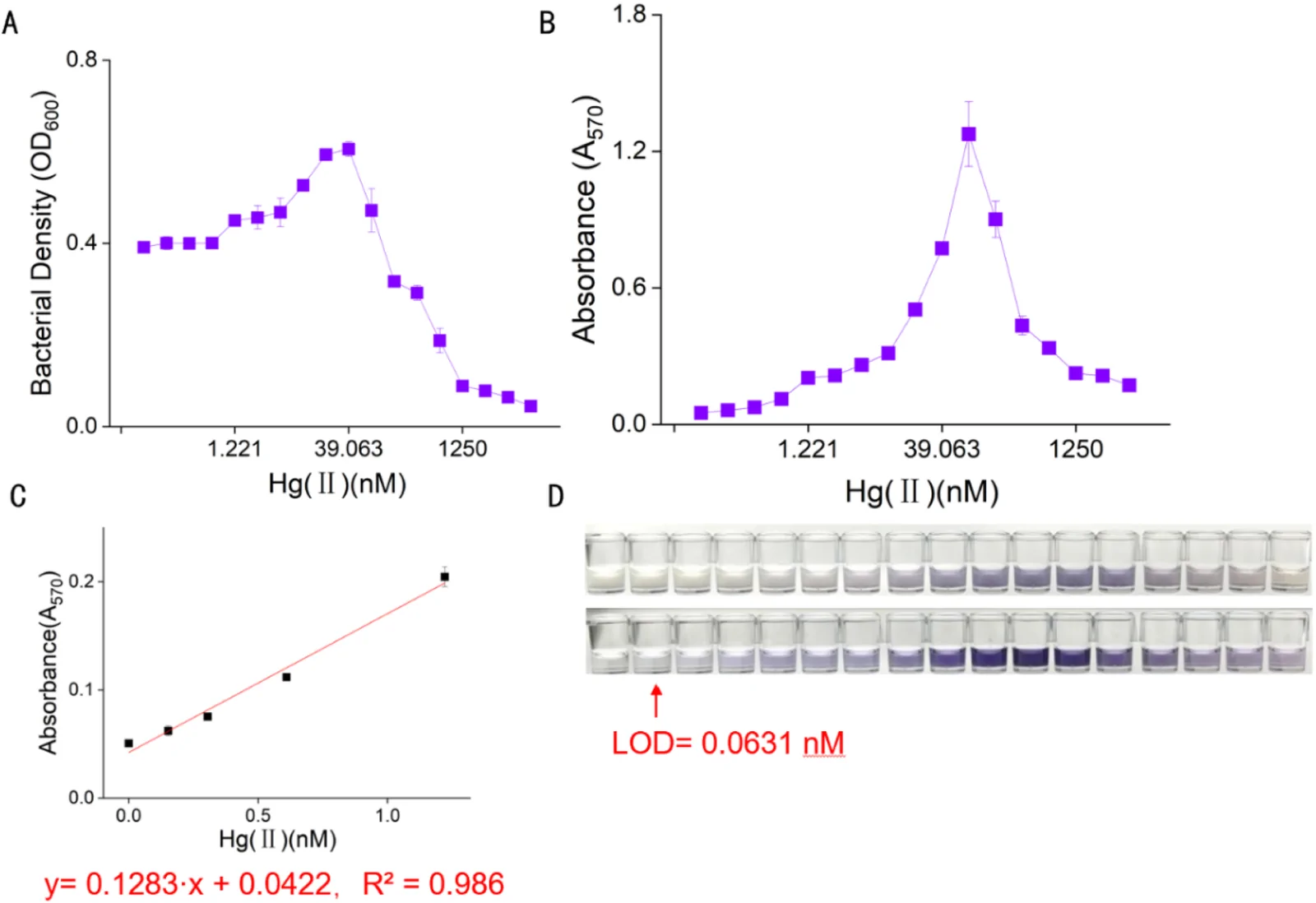
Hg(ii) dose–response profile of the TOP10/pPb-IND-Hg-DV biosensor *via* the DV output channel. (A) Bacterial density (OD_600_) of the Hg(ii) exposure groups. (B) Absorbance response at 570 nm showing DV production across gradient Hg(ii) concentrations. (C) Linear calibration of the low-concentration segment (0–1.221 nM) used for LOD determination. The LOD was calculated as 0.0631 nM. (D) The upper row of cuvettes shows the culture broth, and the lower row shows the *n*-butanol extracts containing DV.

**Fig. 4:**
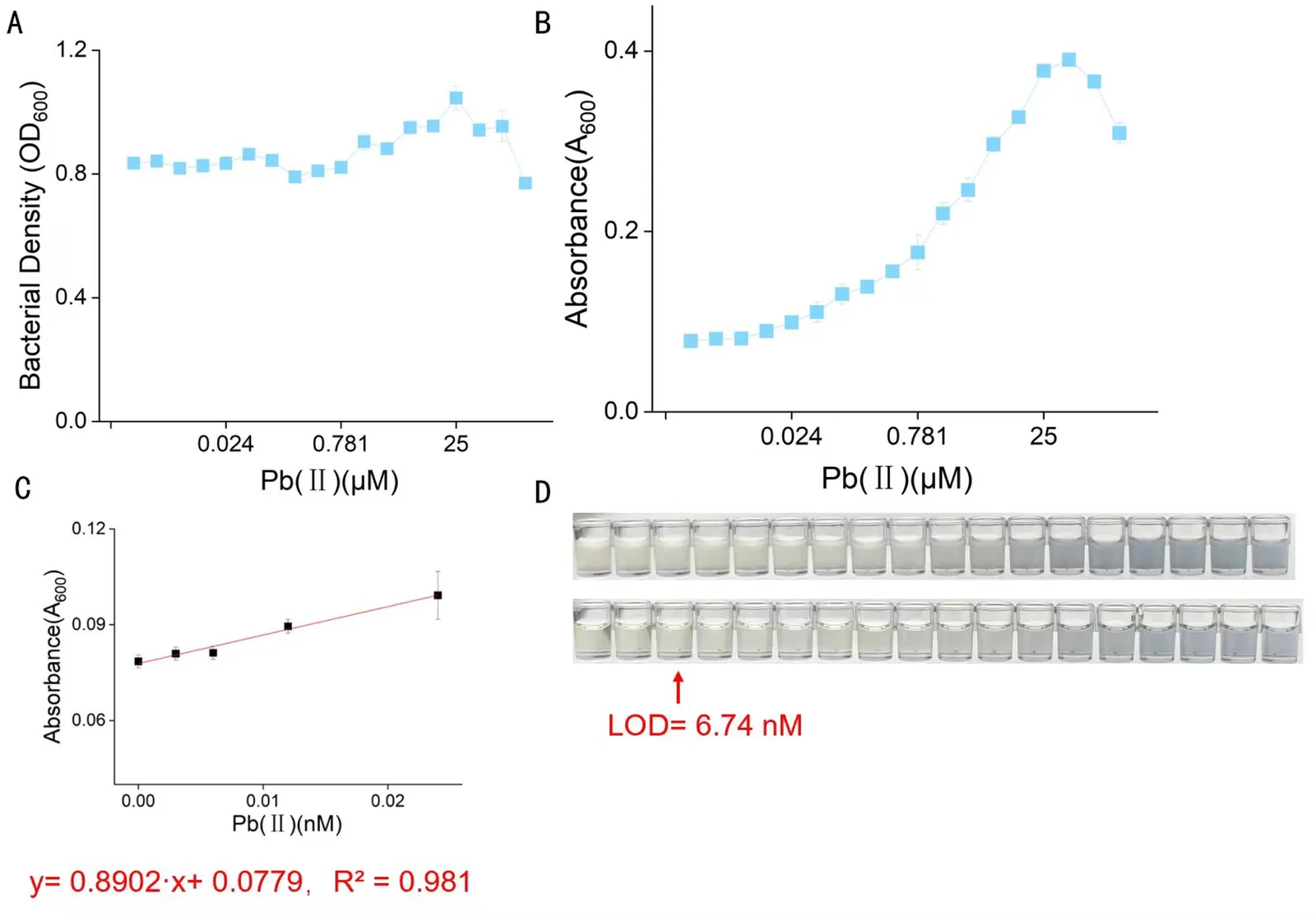
Pb(ii) dose–response profile of the TOP10/pPb-IND-Hg-DV biosensor *via* the IND output channel. (A) Bacterial density (OD_600_) of the Pb(ii) exposure groups. (B) Absorbance response at 600 nm showing IND production across gradient Pb(ii) concentrations. (C) Linear calibration of the low-concentration segment (0–0.024 µM) used for LOD determination. The LOD was calculated as 6.74 nM. (D) The upper row of cuvettes shows the culture broth, and the lower row shows the culture supernatant containing IND.

Instrumental techniques, such as inductively coupled plasma mass spectrometry and atomic absorption spectrometry, remain the gold standard for heavy metal analysis.^34^ However, the associated equipment costs, the need for complex sample preparation, and the requirement for skilled operators restrict their use in large-scale routine surveys and field-based monitoring. In contrast, the microbial biosensor developed here generates quantifiable chromogenic signals through simple cultivation without specialized equipment.^23,24^ Under optimized detection conditions, linear calibration of the low-concentration ascending segments gave, for the Hg(ii)–DV channel, *y* = 0.1283*x* + 0.0422 (*R*^2^ = 0.986; *n* = 5 calibration points over 0–1.221 nM; Fig. 3C) and, for the Pb(ii)–IND channel, *y* = 0.8902*x* + 0.0779 (*R*^2^ = 0.981; *n* = 5 calibration points over 0–0.024 µM; Fig. 4C). The blank standard deviations were *σ* = 0.0027 for the Hg(ii)–DV channel and *σ* = 0.0020 for the Pb(ii)–IND channel, each calculated from three independent blank replicates. Applying LOD = 3*σ*/*k* yielded an LOD of 0.0631 nM for Hg(ii), corresponding to approximately 0.0127 µg L^−1^, and an LOD of 6.74 nM for Pb(ii), corresponding to approximately 1.40 µg L^−1^. The linear fitting plots with equations and *R*^2^ are shown in Fig. 3C (Hg(ii)) and Fig. 4C (Pb(ii)). These values compare favorably with those of previously reported microbial biosensors employing fluorescent protein reporters, which often suffer from narrow dynamic ranges and higher detection limits due to the lack of enzymatic signal amplification.^7^ For instance, our earlier monocistronic IND-based Pb(ii) biosensor reported an LOD of 0.065 µM,^15^ and the DV-based Hg(ii) biosensor achieved an LOD of 0.024 nM.^28^ The present dual-target system, employing decoupled circuits, retains comparable sensitivity while expanding the analytical scope to two metals in a single cellular host.

For quantitative calibration, the low-concentration linear segments used for LOD determination were 0–1.221 nM for the Hg(ii)–DV channel and 0–0.024 µM for the Pb(ii)–IND channel; the broader ascending quantitative working ranges extended to the response peaks at 78.125 nM Hg(ii) and 50 µM Pb(ii), respectively. The exceptionally wide upper limit for Pb(ii) accommodates both trace-level monitoring and assessment of acute pollution events.^35^ The distinct blue and purple pigments permit direct naked-eye observation at environmentally relevant concentrations, making the platform particularly suitable for high-throughput screening of large sample batches and emergency response to pollution incidents where basic sample-processing facilities (*e.g.*, a portable microcentrifuge) are available.

### Phase partitioning of IND and DV and residual cross-channel contribution

To experimentally verify the distribution of IND and DV between the aqueous and *n*-butanol phases, the two pigments were prepared independently using their cognate single-module strains and subjected to a single centrifugation–*n*-butanol extraction identical to the biosensor readout. The absorbance of both phases was recorded at 570 and 600 nm (Fig. S4A). 94.7% of IND remained in the aqueous phase and 5.3% was carried into the *n*-butanol phase, whereas 84.5% of DV partitioned into the *n*-butanol phase and 15.5% remained in the aqueous phase. The apparent partition coefficients, *K*_d_ = Absorbance (*n*-butanol phase)/Absorbance (aqueous phase), were 0.11 for IND and 11.4 for DV, indicating an approximately two-order-of-magnitude difference in phase preference. Since the interference originates from another dye, a cross-correction method was employed. The correction formulas are as follows:IND (true absorbance at 600 nm) = IND (aqueous phase at 600 nm) − 0.0841 × DV (*n*-butanol phase at 570 nm)DV (true absorbance at 570 nm) = DV (*n*-butanol phase at 570 nm) − 0.11 × IND (aqueous phase at 600 nm)

Unless otherwise stated, the absorbance values reported in Fig. 3–8 are raw values; the cross-correction equations above were used to estimate the magnitude of residual crosstalk and to confirm that it does not affect qualitative threshold screening.

**Fig. 5:**
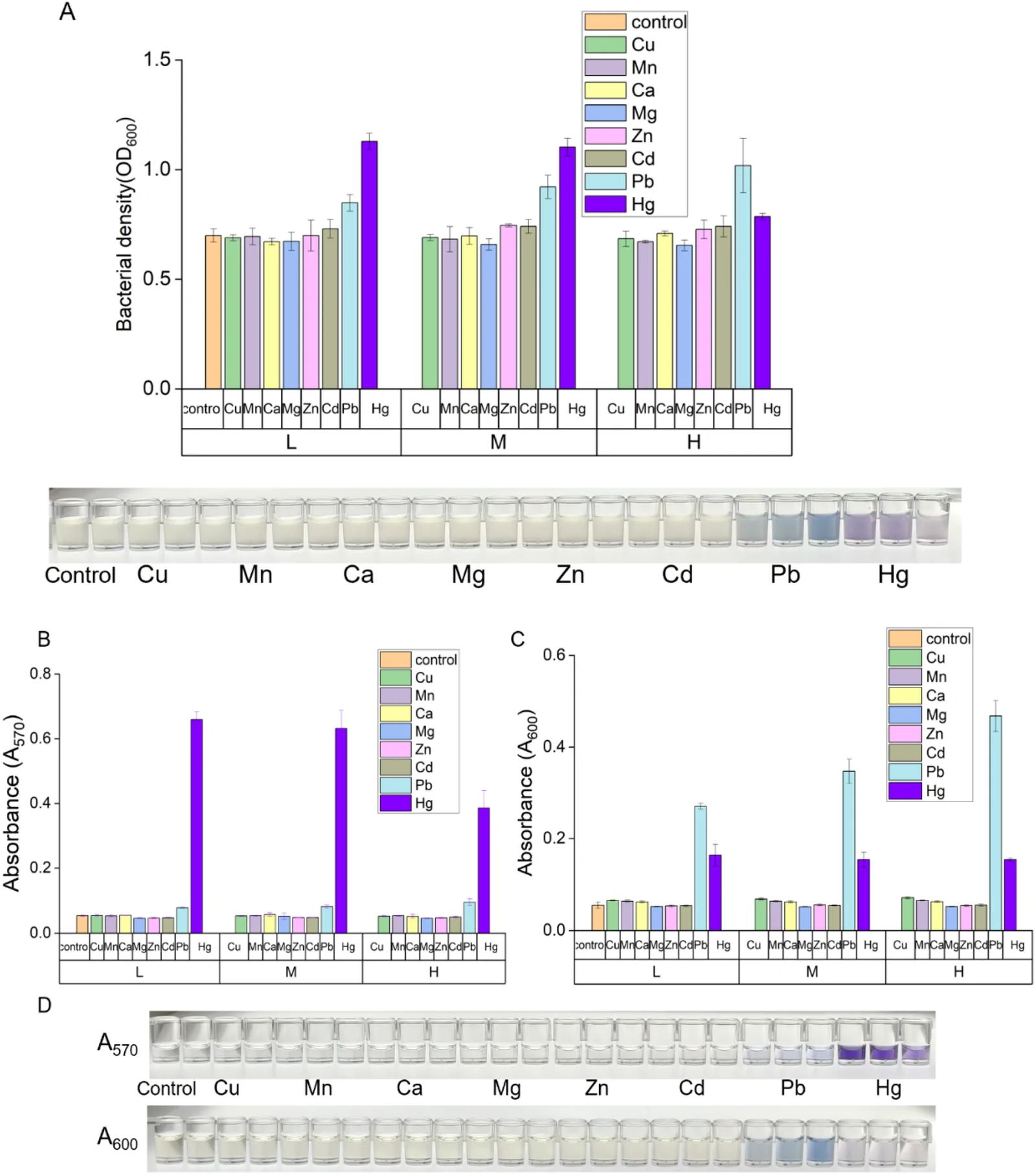
Metal selectivity assessment of the TOP10/pPb-IND-Hg-DV biosensor. (A) Bacterial density (OD_600_) after exposure to individual metal ions at low (L), medium (M), and high (H) concentrations. Hg(ii) was tested at 25, 50, and 100 nM, while all other metals were tested at 2.5, 5, and 10 µM. The lower panel shows the corresponding culture broths. (B) Absorbance of *n*-butanol extracts at 570 nm reflecting the DV signal. (C) Absorbance of culture supernatants at 600 nm reflects the IND signal. (D) Photographs of the *n*-butanol extracts (upper row) and culture supernatants (lower row) for each metal treatment. For each metal, the three cuvettes correspond to low, medium, and high concentrations from left to right.

**Fig. 6:**
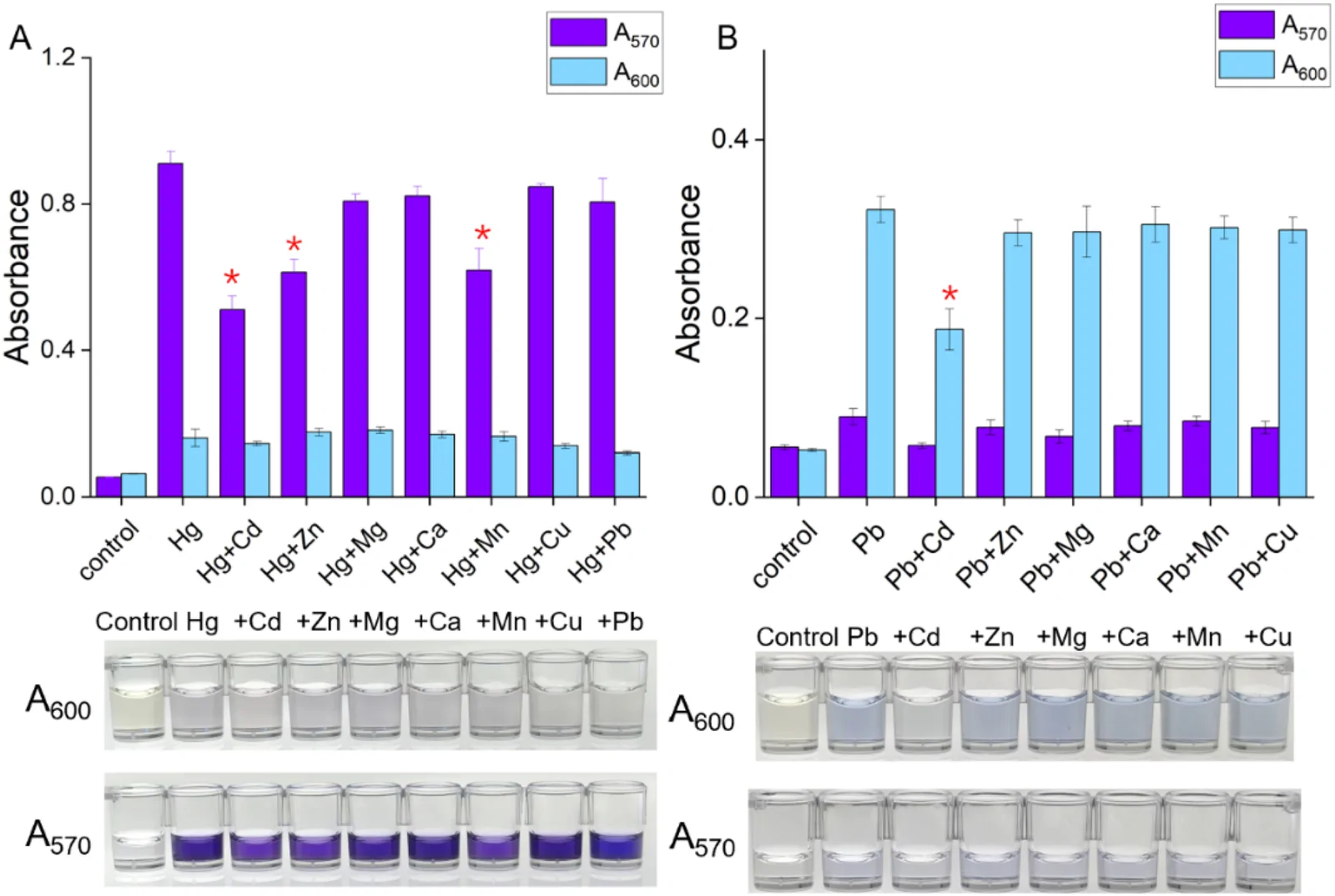
Anti-interference performance of the TOP10/pPb-IND-Hg-DV biosensor. (A) Response signals of 20 nM Hg(ii) co-incubated with various interfering metal ions at 5 µM each. (B) Response signals of 5 µM Pb(ii) co-incubated with various interfering metal ions at 5 µM each. The asterisk indicates a statistically significant difference compared with the group receiving only the target metal. The lower two rows of cuvettes show representative culture supernatants (upper) and *n*-butanol extracts (lower).

**Fig. 7:**
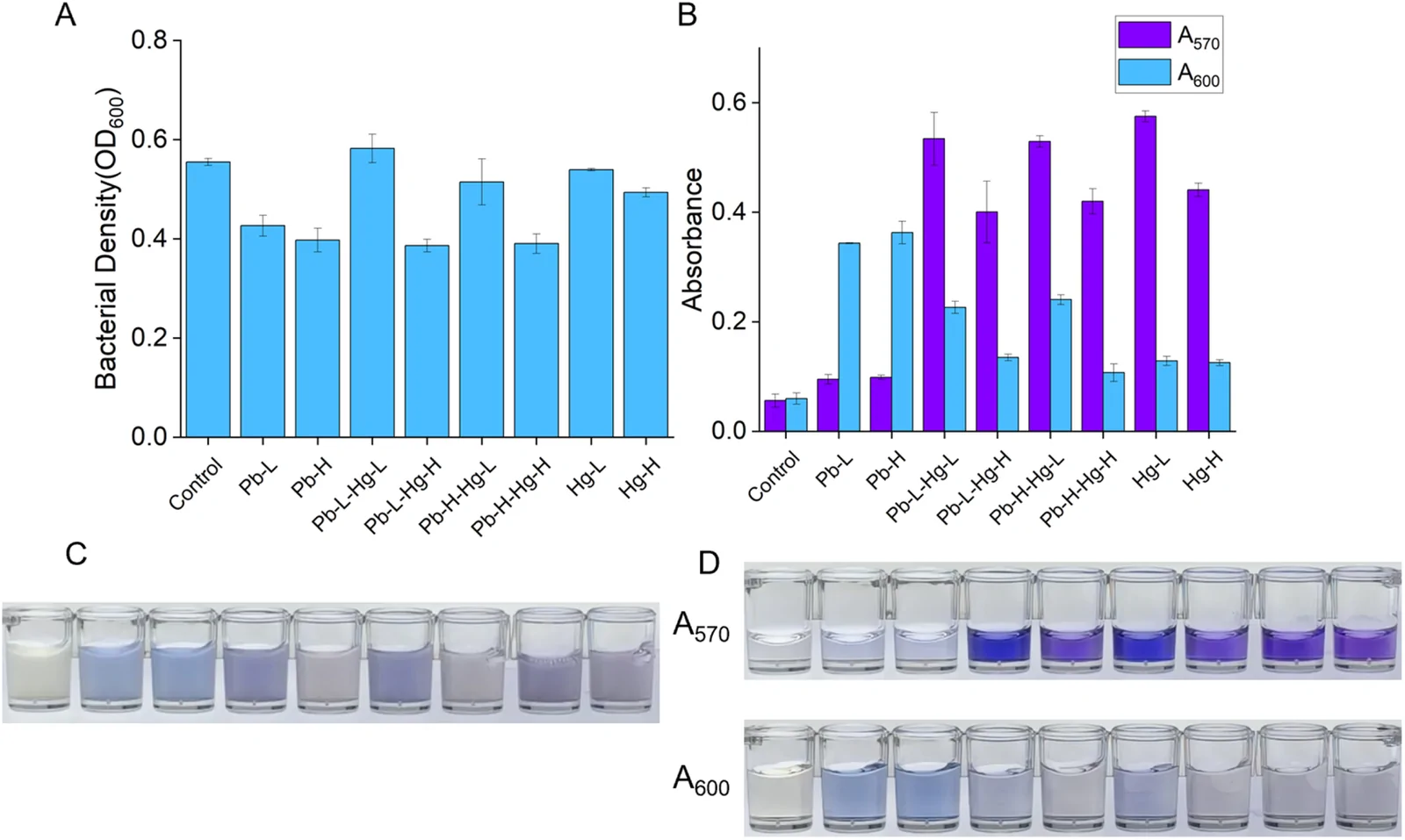
Bacterial growth and dual-pigment signal generation in the presence of individual and mixed Pb(ii) and Hg(ii). (A) Bacterial density (OD_600_) after exposure to varying concentrations of Pb(ii) and Hg(ii). (B) Signal intensities of IND (*A*_600_) and DV (*A*_570_) under the corresponding conditions. (C) Representative photographs of bacterial cultures corresponding to panel A. (D) Representative photographs of *n*-butanol extracts (upper row, containing DV) and culture supernatants (lower row, containing IND) corresponding to panel B. “*L*” denotes the low-concentration group (Pb(ii) = 5 µM, Hg(ii) = 10 nM), and “*H*” denotes the high-concentration group (Pb(ii) = 20 µM, Hg(ii) = 50 nM).

**Fig. 8:**
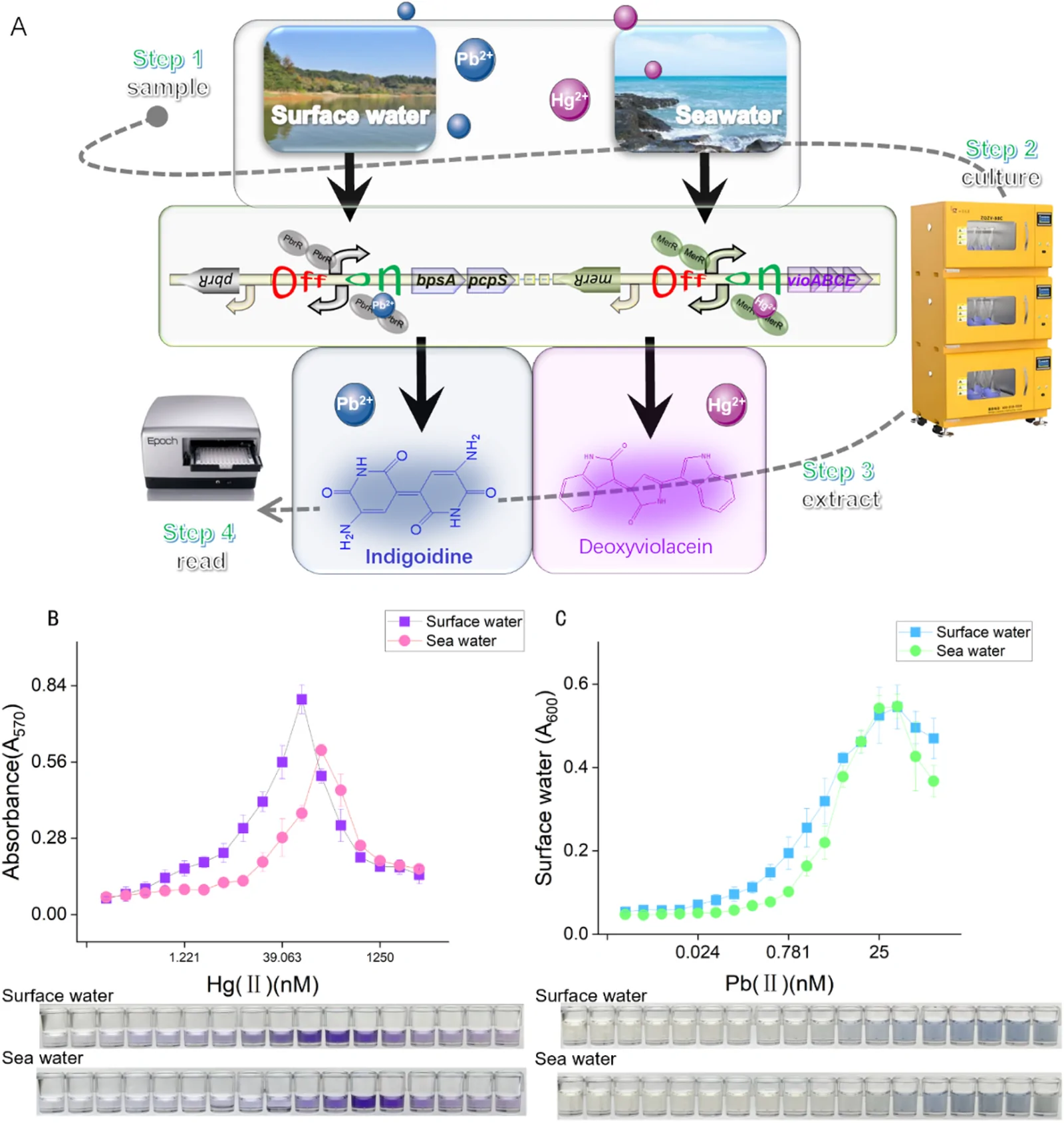
Spiked response curves of the biosensor in seawater and surface water matrices. (A) Schematic workflow of the dual-color whole-cell biosensor for simultaneous detection of Pb(ii) and Hg(ii) in environmental water samples. (B) Full-range absorbance and colorimetric results of the Hg(ii) concentration gradient in surface water and seawater. (C) Full-range absorbance and colorimetric results of the Pb(ii) concentration gradient in surface water and seawater. The microplate photographs below show the corresponding colorimetric gradient responses.

### Metal selectivity of the biosensor

To evaluate the metal response specificity of the dual-target biosensor, TOP10/pPb-IND-Hg-DV was exposed to a panel of divalent metal ions at varying concentrations. Given the pronounced cytotoxicity of Hg(ii), this metal was tested at 25, 50, and 100 nM. Pb(ii) and the remaining ions, including Mg(ii), Ca(ii), Cd(ii), Mn(ii), Cu(ii), and Zn(ii), were applied at 2.5, 5, and 10 µM. All cultures were incubated for 10 h under standardized conditions.

Bacterial growth remained largely unaffected across the tested metal panel, except for a slight turbidity increase in cultures accumulating IND due to the pigmentation of the broth (Fig. 5A). The absorbance signals at 570 nm and 600 nm corresponded to the characteristic maxima of DV and IND, respectively. For the DV channel measured at 570 nm following *n*-butanol extraction, strong signals appeared exclusively in the Hg(ii) treatment groups (Fig. 5B). In contrast, the IND channel measured at 600 nm in the culture supernatant showed pronounced responses only in the Pb(ii) treatment groups (Fig. 5C). Notably, a minor elevation in the 600 nm absorbance was observed in the Hg(ii) groups, which arose from the partial water solubility of DV (15.5% retained in the aqueous phase; Fig. S4) contributing to the supernatant background. This spectral overlap did not compromise the qualitative discrimination, as the *n*-butanol extracts from Hg(ii) cultures turned distinctly purple, whereas the Pb(ii) culture supernatants developed a clear blue color. No statistically significant signals above the blank control were detected for any of the non-target metals in either channel, indicating that the dual regulatory system maintained strict recognition fidelity toward its cognate metal ions.

From a visual inspection standpoint, the color differentiation was unambiguous. The *n*-butanol phase acquired a purple hue exclusively upon exposure to Hg(ii). At the same time, the aqueous supernatant turned blue only upon Pb(ii) exposure (Fig. 5D). The remaining metal ions produced colorless extracts and supernatants indistinguishable from the blank. This dual-channel selectivity confirms that the MerR and PbrR regulatory modules retained their inherent metal recognition properties within the combined genetic architecture, enabling reliable differential detection without cross-activation by competing divalent cations.^22,25^

### Anti-interference performance of the biosensor

#### Anti-interference evaluation of the Hg(ii) response

The sensor cells were co-exposed to 20 nM Hg(ii) together with individual interfering metals at 5 µM each. Coexisting metals did not markedly affect bacterial growth, and the slight variation in OD_600_ was attributable to DV accumulation rather than growth inhibition (Fig. S2A). The IND signals in the culture supernatant at 600 nm remained negligible across all treatment groups, confirming that the Pb(ii) channel was not activated by non-target metals or by the presence of Hg(ii) alone (Fig. 6A). In the DV channel measured at 570 nm following *n*-butanol extraction, all groups containing Hg(ii) showed statistically significant signals compared with the blank control. However, relative to the positive control of Hg(ii) alone, the DV signals in the Hg(ii) + Zn(ii), Hg(ii) + Cd(ii), and Hg(ii) + Mn(ii) groups exhibited a slight decreasing trend. This experiment was performed under deliberately stringent conditions using high concentrations of interfering ions. The attenuation likely reflects competitive binding of these divalent cations to the MerR metal-binding domain, particularly for group 12 elements such as Zn(ii) and Cd(ii), which share chemical similarities with Hg(ii).^36^ Nevertheless, the MerR regulator retains a markedly higher affinity for Hg(ii), and the resulting DV output remained sufficiently robust for qualitative and semi-quantitative detection. These results indicate that the Hg(ii)-DV detection channel maintains acceptable performance even in the presence of competing metal ions.

#### Anti-interference evaluation of the Pb(ii) response

For the Pb(ii) interference experiments, sensor cells were co-exposed to 5 µM Pb(ii) together with individual interfering metals at 5 µM each. Bacterial growth was not significantly affected by the binary combinations, and the increase in OD_600_ was due to IND accumulation in the broth (Fig. S2B). The DV signals in the *n*-butanol extracts showed no difference from the blank control across all treatment groups, confirming minimal leakage from the Hg(ii) channel in the absence of Hg(ii). The IND signals in the culture supernatant at 600 nm were significantly elevated above the background in all groups containing Pb(ii). Compared with the positive control of Pb(ii) alone, only the Pb(ii) + Cd(ii) group showed a modest reduction in IND signal intensity. This finding aligns with previous reports that Cd(ii) can interfere with PbrR-dependent activation of the lead-responsive promoter, likely through competition at the metal-binding cysteine residues of the PbrR dimer.^37^ Despite this slight attenuation, the IND signal remained well above the background level, and the visual blue coloration was clearly preserved. Therefore, the Pb(ii)-IND channel retains reliable detection capability under co-exposure conditions.

### Evaluation of independent channel responses in co-exposure matrices

To rigorously test the biosensor's ability to provide independent quantitative information in the presence of both metal ions, a 3 × 3 cross-concentration matrix assay was conducted. The bacterial density data are shown in Fig. 7A. As shown in Fig. 7B, when the mercury(ii) concentration was fixed (*e.g.*, at 10 nM), the Pb(ii)-induced indigoidine signal (*A*_600_) exhibited a marked dose-dependent increase as the Pb(ii) concentration rose from 0 to 20 µM. Conversely, when the Pb(ii) concentration was fixed at 5 µM, the Hg(ii)-induced DV signal (*A*_570_) was high at low concentrations but decreased at high concentrations. This indicates that a reliable dose–response relationship can be maintained even when the two metals coexist.

The interference caused by the coexistence of the two target ions remained within an acceptable range. The addition of Hg(ii) interferes with the production of a portion of the indigoidine, which is consistent with the selectivity results described earlier. Only when high concentrations of Hg(ii) coexist with low concentrations of Pb(ii) does the indigoidine signal (*A*_600_) show a marked decrease, as the metabolic burden of the DV pigment reduces the production of both pigments; this also aligns very well with the dose–response relationship observed when Hg(ii) is present alone.

Overall, these results do not negate the validity of our system; rather, they objectively reveal the inherent limitations of whole-cell biosensors. Despite these inherent limitations, crosstalk between channels is far lower than the target-specific signal. The system still holds significant practical value for semi-quantitative screening and early warning of complex heavy metal contamination. In practical environmental monitoring, the primary objective is to determine whether regulatory thresholds have been exceeded, rather than to achieve absolute trace-level quantification.

Taken together, the phase-partitioning validation and cross-concentration matrix confirm that the two channels operate with negligible crosstalk under the standard readout protocol.

### Quantitative detection performance in environmental water matrices

Complex matrix components in authentic water samples, including salinity, humic acids, and coexisting ions, can influence the growth, metabolic activity, and specific response of microbial biosensors.^30^ Evaluating sensor performance across diverse water matrices is, therefore, an essential step toward establishing environmental applicability. In this study, surface water was collected from Honghu Park in Luohu District, Shenzhen, and seawater was collected from Dayawan Bay in Yantian District, Shenzhen. All samples were clarified by centrifugation and subsequently sterilized through 0.22 µm membrane filtration to remove suspended particles and indigenous microorganisms while preserving the original dissolved organic and inorganic constituents.^16^ Given the elevated salinity of seawater, a higher dilution ratio was adopted to alleviate salt stress on the engineered host. Following previously established protocols, the surface water detection system was formulated with 90% (v/v) lake water and 10% (v/v) 10× LB, whereas the seawater system contained 50% (v/v) seawater, 40% (v/v) sterile water, and 10% (v/v) 10× NaCl-free LB.^27^ This formulation maintained normal bacterial proliferation and efficient pigment expression while minimizing the elevation of detection limits caused by sample dilution. Dual-channel signal readout was subsequently performed using naked-eye visualization or high-throughput photometry to enable simultaneous screening of heavy metals in both surface water and seawater matrices (Fig. 8A).

TOP10/pPb-IND-Hg-DV was exposed to different concentrations of Pb(ii) or Hg(ii) in both environmental matrices. The sensor demonstrated significant dose–response relationships for both metals in both matrices (Fig. 8B and C); corresponding bacterial density data are shown in Fig. S3. As the concentration of Pb(ii) or Hg(ii) increased, the color of the culture medium in each matrix gradually darkened, and the change was clearly distinguishable to the naked eye. However, there were differences in detection performance between the two matrices: the deviation between the surface water experimental results and the laboratory-grade pure water system was relatively small, whereas the deviation in seawater was larger, with the operating range shifting toward higher concentrations. This indicates that dissolved substances in real-world water bodies—particularly the complex ionic composition of seawater—exert a moderate inhibitory effect on the generation of the chromogenic signal.

According to the Chinese national standard for surface water quality,^38^ the permissible limits for Class III surface water are 50 µg L^−1^ for lead and 0.1 µg L^−1^ for mercury. In comparison, the stricter Class II standards specify 10 µg L^−1^ for lead and 0.05 µg L^−1^ for mercury. The Hg(ii) detection limit of the present biosensor, 0.0631 nM (approximately 0.0127 µg L^−1^), is below the Class II threshold (0.05 µg L^−1^), enabling reliable detection of trace mercury levels that exceed the regulatory limit. The Pb(ii) detection limit of 0.0067 µM (6.74 nM, approximately 1.40 µg L^−1^) is below the Class II requirement (10 µg L^−1^ for lead) and well below the Class III limit (50 µg L^−1^), supporting rapid screening of lead-contaminated water and emergency response to pollution incidents. Furthermore, the upper quantitative limits of the biosensor reach 15.7 µg L^−1^ (78 nM) for Hg(ii) and 10.4 mg L^−1^ (50 µM) for Pb(ii), which cover the concentration ranges typically required for routine water quality monitoring and pollution event assessment.^4,39^ These characteristics indicate that the dual-color biosensor holds considerable promise for on-site environmental screening and rapid response to heavy metal pollution incidents.

The 10 h incubation period of the present whole-cell biosensor is inherently longer than the minute-scale responses reported for fluorescent small-molecule probes, DNAzyme-based sensors, and protein-based fluorescent biosensors.^40–42^ However, these faster methods generally require excitation sources, emission filters, controlled dark-room conditions, and trained operators to mitigate photobleaching and background interference.^40,41^ Protein-based fluorescent sensors, while highly sensitive, also rely on sophisticated optical readers and refrigerated reagents, restricting their use in resource-limited field settings.^42^ In contrast, the pigment-based colorimetric output described here is read in a standard microplate format using ambient light and simple absorbance photometry—or, for threshold screening, by the naked eye without any instrument. Consequently, the longer incubation represents a deliberate trade-off: speed is sacrificed for instrumental minimalism, low per-assay cost, and visual discriminability, attributes that are particularly advantageous for on-site environmental monitoring and emergency pollution assessment where laboratory infrastructure is unavailable.

The current workflow requires a single 10 min centrifugation step (12 000×*g*) and a one-time *n*-butanol extraction to separate the aqueous IND signal from the organic DV signal. These steps, while routine in any minimally equipped laboratory or mobile testing unit, pose a practical constraint to completely instrument-free field deployment. We therefore position the present biosensor as a preliminary screening and early-warning tool that complements—not replaces—definitive instrumental analysis (*e.g.*, ICP-MS). Future engineering efforts, such as lyophilized whole-cell paper strips or integrated microfluidic devices with built-in phase separation, could eliminate the need for centrifugation and bring the platform closer to true point-of-use applicability.

## Conclusions

This study presents a dual-color whole-cell biosensor that enables simultaneous visual detection of Pb(ii) and Hg(ii) within a single bacterial host. By integrating a Pb(ii)-responsive PbrR module that drives IND biosynthesis with an Hg(ii)-responsive MerR module that drives DV biosynthesis, the engineered sensor generates distinct blue and purple signals quantifiable at 600 nm and 570 nm, respectively. The system achieved limits of detection of 0.0067 µM (6.74 nM) for Pb(ii) and 0.0631 nM for Hg(ii), maintained broad dynamic ranges, and performed robustly in spiked surface water and seawater matrices. Compared with our earlier dual-color biosensor that employed PDV as the Pb(ii) reporter, the present IND/DV architecture represents a conceptual redesign rather than a simple pigment substitution. Replacing the oxidation-dependent, organic-phase PDV with the water-soluble, chemically stable IND eliminates the post-cultivation oxidation step, enables automatic aqueous/organic-phase separation of the two readouts, and provides a more discriminable blue-*versus*-purple color pair for naked-eye screening. These improvements yield a more field-compatible platform for preliminary environmental monitoring and emergency pollution assessment.

## Author contributions

Zongjie Ma: writing – original draft, investigation, formal analysis. Yan Guo: funding acquisition, writing – review and editing. Wenjun Luo, Keyan Wei, Zhaobo Hu, Jiajun Huang: investigation. Yuting Chen: resources. Chang-ye Hui: writing – original draft, project administration, funding acquisition, conceptualization.

## Conflicts of interest

There are no conflicts to declare.

## Supplementary Material

RA-OLF-D6RA05888D-s001

## Data Availability

The data that support the findings of this study are available on reasonable request from the corresponding author. Supplementary information (SI): the DNA sequence of the *E. coli* TOP10/pPb-IND-Hg-DV sensor used in this study (Table S1); the full-wavelength absorption spectra of the two pigments (Fig. S1); the bacterial density that reflects the sensor's resistance to interference from coexisting metal ions. (Fig. S2); bacterial growth density corresponding to the sensor's response to mercury and lead ions in environmental matrices (Fig. S3); and an assessment of the partitioning of the two pigments between the detection channels and residual interference (Fig. S4). See DOI: https://doi.org/10.1039/d6ra05888d.
