# Supplementary material for: Indigoidine and deoxyviolacein co-pigmentation in a single bacterial cell enables visual discrimination of Pb(ii) and Hg(ii) in environmental water matrices
Source: RSC Adv. 2026 Oct 19;16(48):64452–64. doi: 10.1039/d6ra05888d (PMC13679184; doi:10.1039/d6ra05888d)
Supplement: Supplementary file 1 [file RA-016-D6RA05888D-s001.pdf]

Supplementary Information (SI) for RSC Advances.
This journal is © The Royal Society of Chemistry

Supplementary materials

Table S1. DNA sequence of the sensing plasmid pPb-indigoidine-Hg-DV used in this study

DNA sequence of the Pb(II) and Hg(II) dual-target sensing module inserted into the pPb-indigoidine-Hg-DV plasmid
AGATCTTTACCCAGATGTTTGACTGTTCGTGGCACTTTCACCATGGCAATTGCCCAACCCTTGCAAAATGCCGCA<br>CGCCTCTACAGATCGAGAGCCAGAACACTTCTCGCGCAAATCAACCAAGTGCCGTTTTAACTGCAACAGCGCGG<br>ACACACGCATTTCCACCTGTTGAATATGGGCCTCCAGCAGCGTGATGACCTCCCCACAGTCCTGCATCGGGTTG<br>TCTCGCAGACCCAGCAATGCGCGAATCTCGCTCAACGTCATGTCGAGCGAACGGCAATGACGGATGAATTGCAA<br>GCGCTCAATGTGCGCCTCACCGTACAACCGAAAGTTGCCACCGCTTCGCGCTGGCTTTGGCAGTAGCCCTTCCT<br>TCTCGTAGTAGCGGATGGTCACGACCTCGCACCCAGAGCGCTTGGCGAGGTCGCCAATTCTGATTTCCATCGTT<br>CGCGCAACGCCTCCTGGTACCTGTGCTTTGTTAAAGGGTTTCAACAGATATTCAAGCATCAATCTCCAATTATCA<br>CTTGACTCTATAGTGACTATAGAGATTTTAATGGAGGCTGAATAGAAGATTTTCAGGAGTTACTCTCTAGAAATA<br>ATTTTGTTTAACTTTAAGAAGGAGATATACATATGACCCTGCAGGAAACCTCTGTTCTGGAACCGACCCTGCAGG<br>GCACCACCACGCTGCCGGGTCTGCTGGCTCAGCGCGTAGCTGAACACCCGGAAGCTATCGCAGTTGCTTACCGT<br>GATGACAAACTGACCTTCCGTGAACTGGCTTCTCGCTCGGCCGCGCTGGCGGACTACCTGGAACACCTGGGTGT<br>CTCCGCGGACGACTGTGTTGGTCTGTTCGTAGAACCGTCTATCGACCTGATGGTTGGCGCGTGGGGCATCCTGA<br>ACGCCGGCGCGGCATACCTGCCGCTGAGCCCGGAATACCCGGAAGATCGCCTGCGTTACATGATCGAAAACAGC<br>GAAACTAAAATTATCCTGGCCCAGCAGCGCCTGGTTAGCCGCCTGCGTGAACTCGCCCCGAAAGACGTTACCAT<br>TGTTACTTTACGTGAATCTGAAGCCTTTGTGCGTCCGGAAGGCACGGAAGCGCCGGCGGCACGCTCCGCTCGTC<br>CGGACACCCTGGCCTACGTGATCTATACTTCCGGTAGCACCGGAAAACCGAAAGGTGTTATGATTGAACACCGC<br>TCTATCGTTAACCAACTGGGCTGGCTGCGTGAAACCTACGCCATCGACCGTTCTAAAGTAATTCTGCAGAAAACT<br>CCGATGTCGTTCGACGCTGCTCAGTGGGAAATTCTGTCTCCGGCTAACGGCGCGACCGTTGTTATGGGCGCACC<br>GGGTGTCTACGCGGACCCGGAAGGTCTGATCGAAACCATCGTGAAACATAACGTTACCACTCTGCAGTGCGTTC<br>CGACCCTGCTGCAGGGTCTGATTGATACCGAGAAATTCCCGGAATGCGTAAGTCTGCAGCAGATTTTCTCCGGC<br>GGTGAAGCGCTGTCCCGCCTGCTGGCCATCCAGACCACTCAAGAAATGCCGGGCCGTGCCCTGATCAACGTTTA<br>CGGCCCTACAGAAACCACGATTAACAGCTCCTCTTTTCCGGTAGATCCGGCAGACCTCGATGAAGGCCCGCAGA<br>GCATCAGCATCGGTAGCCCGGTACACGGTACCACCTACCACATCCTGGACAAAGAAACCCTCAAACCGGTCGGC<br>GTCGGCGAAATTGGTGAACTGTATATCGGCGGCATTCAGCTGGCTCGCGGCTACCTGCACCGTGACGACCTGAC<br>TGCCGAACGCTTCCTGGAGATTGAACTGGAAGAAGGTGCGGAACCGGTTCGCTTGTATAAAACTGGTGATCTGG<br>GTCAGTGGAACAACGATGGCACGGTTCAGTTCGCTGGTCGTGCAGACAACCAAGTGAAACTGCGTGGTTATCGC<br>GTTGAACTTGATGAAATTAGCCTGGCGATTGAAAATCACGATTGGGTTCGTAACGCTGCAGTGATCGTGAAAAAT<br>GACGGTCGTACTGGTTTTCAGAACCTGATCGCTTGTATTGAACTGAGTGAAAAAGAAGCAGCACTGATGGATCA<br>GGGTAATCATGGTTCACATCATGCTTCTAAAAAATCCAAACTGCAGGTGAAGGCCCAGCTGAGCAATCCGGGTC<br>TGCGTGATGATGCGGAATTAGCCGCTCGCCCGGCCTTCGACCTGGAAGGTGCAGAGCCAACCCCTGAACAACGT<br>GCGCGTGTTTTTGCGCGCAAAACGTACCGTTTCTACGAGGGCGGCGCGGTGACGCAGGCTGACTTGCTGGGCC<br>TGCTGGGTGCGACCGTGACCGCGGGTTATTCTCGTAAAGCTGCCGATCTGGCACCGGCGGAACTGGGCCAGAT<br>TCTGCGTTGGTTCGGCCAGTACATTTCTGAAGAACGTCTGTTACCAAAATATGGTTACGCTTCTCCTGGTGCTCT<br>GTACGCAACCCAAATGTACTTCGAACTGGAAGGTGTTGGCGGTTTGAAACCGGGTTATTATTACTACCAACCGG<br>TTCGTCACCAGCTCGTGTTGATTTCTGAACGTGAAGCCACCGGTAAAGCCACCGCACAGATCCACTTCATTGGC<br>AAAAAGAGTGGCATTGAACCGGTTTATAAAAATAACATCTTGGAAGTTCTTGAAATTGAAACTGGTCACATGGTT<br>GGTCTGTTCGAACAGATCCTGCCGGCATACGGCCTCGATATTCATGATCGTGCGTACGAACCGGCTGTTAAGGA<br>TCTGCTGGACGTTGCGGATGAGGACTACTACCTGGGCACTTTCGAACTGGTTCCGCACGCGGGTGCACGCGAC<br>GATCAGGCGGAAGTCTATGTACAGACCCACGGTGGCAAAGTAGCTGGTCTGCCGGAAGGTCAGTATCGCTATGA<br>AAATGGTGAGTTGACTCGCTTCAGCGACGACATTGTTCTGAAAAAACACGTTATTGCTATCAATCAGTCCGTCTA<br>CCAAGCTGCGTCCTTTGGTATCAGCGTTTACTCGCGTGCAGAAGAAGAATGGTTAAAGTACATCACGCTGGGTA<br>AAAAACTGCAACATCTGATGATGAACGGTCTTAACCTGGGCTTTATGTCCAGCGGTTACTCCTCTAAAACCGGTA<br>ATCCGCTGCCGGCGTCCCGCCGTATGGATGCTGTTCTGGGTGCTAACGGCGTGGACAGCGCGCCAATGTATTTC<br>TTTGTTGGTGGCCGCATTTCGGACGAACAGATCGGTCACGAAGGTATGCGTGAAGATTCCGTGCACATGCGCGG<br>TCCGGCTGAACTGATCCGTGACGACCTGGTTAGCTTCCTGCCGGACTATATGATCCCGAACCGTGTCGTGGTAT<br>TCGATCGTCTGCCACTGAGCGCGAACGGTAAAATCGACGTTAAAGCACTGGCGGCCTCTGATCAGGTGAACGCG<br>GAGCTGGTTGAACGTCCGTTTGTCGCACCGCGTACTGAAACAGAAAAAGAAATTGCTGCTGTGTGGGAAAAAGC<br>ACTGCGTCGTGAAAACGCCTCTGTGCAAGATGACTTCTTTGAGAGCGGCGGGAACTCCCTGATTGCGGTTGGTC<br>TGGTTCGTGAACTGAACGCGCGTTTAGGCGTCAGCTTACCGCTGCAGTCTGTACTGGAGTCCCCGACCATTGAA<br>AAACTGGCACGTCGCCTGGAACGTGAAGTGGCGCAGGAATCAAGCCGTTTTGTTCGTTTACACGCAGAAACTGG<br>TAAAGCGCGTCCGGTGATTTGCTGGCCGGGCCTGGGCGGTTATCCTATGAACCTGCGTAGCCTGGCAGGTGAAA<br>TCGGCCTGGGCCGTAGCTTCTATGGTGTGCAGAGCTACGGCATTAACGAAGGTGAAACCCCGTATGAAACTATC<br>ACCGAGATGGCGAAAAAGATATCGAGGCTCTGAAAGAAATCCAGCCGGCGGGGCCGTATACCCTGTGGGGCTA<br>CTCTTTCGGTGCGCGCGTTGCGTTTGAAACCGCCTATCAGCTGGAACAGGCTGGTGAAAAAGTGGATAACCTGT<br>TCCTGATTGCACCGGGCTCACCGAAAGTTCGCGCTGAGAACGGTAAAGTTTGGGGTCGCGAAGCGAGCTTCGCA<br>AACCGCGGATACACGACCATCCTGTTTTCTGTGTTTACTGGCACCATTTCCGGCCCAGACCTGGACCGTTGCCT<br>GGAAACCGTGACTGATGAAGCATCCTTCGCTGAGTTCATCTCAGAGCTGAAAGGTATTGACGTTGACCTGGCGC

GTCGCATCATCAGCGTAGTGGGCCAGACGTACGAGTTCGAATACTCTTTCCATGAACTGGCGGAGCGTACCCTG
CAGGCGCCGATCTCTATCTTCAAAGCCGTGGGCGATGACTACTCCTTCCTGGAAAATAGCTCTGGCTATTCCGC
GGAACCGCCGACCGTGATCGATCTGGATGCGGATCATTACAGCCTGCTGCGTGAAGATATCGGTGAACTGGTAA
AACATATCCGCTATCTCCTGGGTGAATAATTAAGGAGGTAAAAAAAATGCGTGCGATGAACGATCGTCTGCCGT
CCTTCTGCACCCCGCTGGATGATCGTTGGCCGCTGCCGGTTGCGCTGCCGGGCGTGCAGCTGCGTTCGACCCG
CTTCGATCCGGCGCTGCTGCAGCCGGGCGATTTCGCGCTGGCGGGCATCCAGCCGCCGGCGAACATCCTGCGT
GCGGTGGCGAAACGTCAGGCGGAGTTCCTGGCGGGTCGTCTGTGCGCGCGTGCGGCGCTGTTCGCGCTGGATG
GTCGTGCGCAGACTCCGGCTGTTGGCGAAGATCGTGCGCCGGTTTGGCCGGCGGCGATCTCCGGCTCTATCAC
CCACGGCGATCGCTGGGCGGCGGCGCTGGTTGCGGCGCGCGGCGATTGGCGTGGTCTGGGTCTGGATGTTGAA
ACCCTGCTGGAAGCGGAACGTGCGCGTTACCTGCACGGCGAAATCCTGACCGAAGGCGAACGTCTGCGTTTCGC
GGATGATCTGGAACGTCGTACCGGCCTGCTGGTGACCCTGGCGTTCTCCCTGAAAGAAAGCCTGTTCAAAGCGC
TGTACCCGCTGGTTGGCAAACGTTTCTACTTCGAACACGCAGAACTGCTGGAATGGCGTGCGGACGGTCAAGCG
CGTCTGCGTCTGCTGACCGATCTGAGCCCGGAATGGCGTCACGGTTCTGAACTGGATGCGCAGTTCGCGGTTCT
GGACGGCCGTCTGCTGTCTCTGGTTGCGGTTGGTGCGTAAgagctcCTAAGGCATAGCTGACCTTGCCAGGCCTGC
TTCGCCCTGTAGTGACGCGATCAACGGGCAGGAAACATTCCCCTTTCGTGCATGGCAGGCGCACACGAGTTCAG
ACAGCACGGTTTCCATGCGCGCCAAGTCGGCCATCTTCTCGCGCACGTCCTTGAGCTTGTGTTCGGCCAGGCTG
CTGGCCTCCTCGCAGTGGGTGCCATCGTCGAGCCGCAACAGCTCGGCAATCTCGTCCAGACTGAACCCCAGCCG
CTGTGCCGATTTCACGAATTTCACCCGAACCACGTCCGCCTCCCCATAGCGGCGGATGCTGCCGTAAGGCTTGT
CCGGTTCCCGCAACAGGCCCTTGCGCTGATAGAAGCGGATTGTCTCCACGTTGACCCCGGCCGCCTTGGCAAAA
ACGCCAATGGTCAGGTTTTCCAAATTATTTTCCATCGTTCGCGCAACGCCTCCTGGTACCTGTGCTTTGTTAAAG
GGTTTCAACAGATATTCAAATCGCTTGACTCCGTACATGAGTACGGAAGTAAGGTTACGCTATCCAATCCAAATT
CAAAAGGGCCAACGTTCTAGAAATAATTTTGTTTAACTTTAAGAAGGAGATATACATATGAAACACAGCTCTGAT
ATCTGCATCGTTGGTGCGGGCATCTCTGGTCTGACCTGCGCGAGCCACCTGCTGGATTCTCCGGCGTGCCGTG
GTCTGAGCCTGCGTATCTTCGATATGCAGCAGGAAGCGGGTGGCCGTATCCGTTCTAAAATGCTGGATGGTAAA
GCGAGCATTGAACTGGGTGCGGGTCGTTACAGCCCGCAGCTGCACCCGCACTTCCAGTCCGCTATGCAGCACTA
CAGCCAGAAATCCGAAGTTTACCCGTTTACCCAGCTGAAATTCAAATCTCACGTTCAGCAGAAACTGAAACGCGC
GATGAACGAACTGTCTCCGCGTCTGAAAGAACACGGTAAAGAATCTTTCCTGCAGTTCGTTTCCCGTTACCAGG
GCCACGATAGCGCGGTTGGCATGATCCGTTCTATGGGTTACGATGCGCTGTTCCTGCCGGATATCTCTGCGGAA
ATGGCGTATGATATCGTTGGTAAACACCCGGAAATCCAGAGCGTTACCGATAACGATGCGAACCAGTGGTTCGC
GGCGGAAACCGGTTTCGCGGGTCTGATCCAGGGCATCAAAGCGAAAGTAAAAGCTGCTGGTGCACGTTTCTCTC
TGGGTTACCGTCTGCTGAGCGTTCGCACCGATGGCGATGGTTACCTGCTGCAGCTGGCGGGCGACGATGGCTG
GAAACTGGAACACCGTACCCGTCACCTGATCCTGGCGATCCCGCCGTCTGCGATGGCGGGCCTGAACGTTGATT
TCCCGGAAGCGTGGTCCGGCGCGCGTTACGGTTCTCTGCCGCTGTTCAAAGGTTTCCTGACCTACGGTGAACCG
TGGTGGCTGGATTATAAACTGGATGATCAGGTGCTGATCGTTGATAACCCGCTGCGTAAAATCTACTTCAAAGG
TGATAAATACCTGTTCTTCTACACTGATAGCGAAATGGCTAACTACTGGCGTGGTTGCGTTGCTGAAGGTGAAG
ATGGTTACCTGGAACAGATCCGTACCCACCTGGCGTCTGCTCTGGGCATCGTTCGTGAACGTATCCCGCAGCCG
CTGGCGCACGTTCACAAATACTGGGCGCACGGCGTTGAATTTTGCCGTGATAGCGATATTGATCACCCGTCCGC
GCTGAGCCACCGTGATTCTGGTATCATCGCTTGCTCTGATGCTTACACCGAACACTGCGGCTGGATGGAAGGCG
GTCTGCTGAGCGCGCGTGAAGCGAGCCGTCTGCTGCTGCAGCGTATCGCGGCGtaattaaggaggtaaaaaaaatgAGCATCCT
GGATTTCCCGCGTATCCATTTCCGTGGCTGGGCGCGTGTTAACGCCCCGACTGCGAACCGTGACCCGCACGGC
CACATTGATATGGCGTCTAATACCGTCGCAATGGCGGGTGAACCGTTCGATCTGGCGCGCCACCCGACCGAATT
TCACCGTCACCTGCGTTCTCTGGGCCCGCGTTTCGGCCTGGATGGCCGTGCAGACCCGGAAGGCCCGTTCAGC
CTGGCGGAAGGCTATAACGCAGCGGGTAACAACCATTTTTCTTGGGAATCTGCGACCGTGTCTCATGTGCAGTG
GGATGGCGGCGAAGCAGATCGTGGTGACGGTCTGGTCGGCGCTCGCCTGGCGCTGTGGGGTCACTATAACGAC
TACCTCCGTACCACCTTCAACCGTGCGCGTTGGGTGGACTCTGACCCGACACGTCGTGATGCGGCCCAGATCTA
CGCAGGTCAATTCACTATCAGCCCGGCTGGTGCGGGCCCTGGCACCCCGTGGCTGTTCACTGCGGATATCGAC
GACTCCCACGGCGCGCGCTGGACCCGTGGTGGCCACATCGCAGAACGCGGTGGCCATTTCCTGGACGAAGAGT
TCGGCCTGGCGCGTCTGTTCCAATTTAGCGTGCCAAAAGATCATCCGCACTTCCTGTTCCATCCGGGGCCGTTC
GATTCTGAGGCATGGCGTCGTCTGCAGCTGGCTCTGGAGGACGATGATGTGCTGGGTCTGACCGTTCAATACGC
TCTGTTCAACATGAGCACTCCGCCGCAGCCGAACAGCCCGGTGTTCCATGATATGGTCGGTGTTGTTGGCCTGT
GGCGCCGTGGTGAACTGGCCAGCTACCCGGCCGGCCGTCTGCTGCGTCCGCGCCAGCCGGGTCTGGGTGATCT
GACCCTTCGTGTTAACGGTGGCCGCGTGGCCTTGAACCTGGCTTGCGCGATCCCGTTCTCTACTCGCGCGGCTC
AGCCGAGCGCACCGGATCGCCTGACCCCGGATCTGGGCGCGAAACTGCCGCTGGGTGATCTCCTGCTGCGTGA
TGAAGATGGTGCACTGCTGGCACGCGTTCCGCAGGCGCTCTACCAGGACTACTGGACTAACCACGGCATTGTGG
ATCTGCCGCTGCTGCGTGAGCCGCGTGGTTCCTTAACCCTGTCCTCTGAACTGGCCGAGTGGCGTGAACAGGAC
TGGGTGACGCAGAGTGATGCTTCCAACCTGTATCTGGAGGCTCCGGACCGCCGTCATGGCCGTTTCTTCCCGGA
ATCCATTGCTCTGCGTTCTTACTTTCGCGGCGAAGCACGCGCTCGCCCGGATATCCCACACCGTATTGAAGGCA
TGGGTCTGGTAGGTGTTGAATCCCGCCAAGACGGCGATGCGGCGGAATGGCGTCTGACCGGCCTGCGTCCTGG
TCCTGCACGCATTGTGCTCGATGATGGTGCGGAAGCAATCCCGCTGCGTGTCCTGCCGGATGATTGGGCGCTG
GATGACGCAACGGTGGAAGAAGTAGACTACGCGTTCCTGTACCGCCACGTGATGGCGTACTATGAGTTGGTCTA
TCCGTTCATGAGCGATAAAGTTTTCAGCCTGGCGGACCGTTGTAAATGCGAAACCTATGCTCGTCTGATGTGGC
AGATGTGCGATCCTCAGAACCGTAATAAATCTTACTATATGCCGTCCACGCGCGAACTGAGCGCGCCGAAAGCA
CGCTTGTTTCTGAAATACCTGGCGCATGTAGAAGGTCAGGCACGCCTCCAGGCTCCGCCGCCGGCGGGCCCGG
CCCGCATCGAAAGCAAAGCTCAGCTGGCCGCCGAACTGCGTAAAGCAGTTGACCTGGAACTGTCCGTCATGCTG
CAATATCTCTACGCTGCATATAGCATCCCAAACTACGCGCAAGGTCAGCAGCGCGTGCGTGATGGCGCGTGGAC
CGCTGAACAGCTGCAGCTGGCATGTGGTAGCGGCGATCGCCGCCGCGACGGCGGTATCCGCGCGGCGCTGCTG
GAGATTGCGCACGAAGAGATGATCCACTATCTGGTCGTGAACAATCTGCTGATGGCCCTGGGCGAACCGTTCTA

CGCGGGCGTTCCTCTGATGGGTGAAGCGGCACGTCAGGCGTTTGGCCTGGACACTGAGTTCGCCCTGGAGCCG
TTTTCTGAATCTACTCTGGCGCGCTTCGTTCGCCTGGAATGGCCGCATTTCATCCCGGCGCCGGGCAAGTCCAT
CGCTGACTGTTACGCAGCAATTCGCCAGGCATTCCTGGACCTGCCGGACCTGTTTGGTGGCGAAGCCGGTAAAC
GTGGTGGCGAACACCACTTGTTTCTGAATGAACTGACCAATCGCGCGCATCCGGGTTACCAGCTGGAAGTGTTT
GATCGTGATTCCGCCCTGTTTGGTATTGCGTTCGTGACCGATCAGGGCGAAGGTGGTGCTCTGGATTCCCCGCA
CTACGAACATAGCCACTTTCAGCGCCTGCGTGAAATGTCGGCTCGTATCATGGCGCAAAGCGCTCCATTCGAAC
CGGCGCTGCCGGCCCTGCGTAACCCGGTCCTGGATGAGTCTCCGGGCTGCCAGCGTGTTGCGGATGGTCGTGC
GCGTGCGCTGATGGCGCTGTATCAGGGTGTTTACGAACTGATGTTCGCGATGATGGCACAGCATTTTGCTGTTA
AACCGCTGGGTTCTCTGCGTCGTAGCCGCCTGATGAACGCTGCAATCGATCTGATGACCGGTCTGCTGCGTCCG
CTGAGCTGCGCCCTGATGAATCTGCCGAGCGGTATTGCGGGTCGTACCGCGGGTCCGCCGCTGCCTGGCCCGG
TTGACACTCGTTCCTATGATGACTACGCACTGGGCTGCCGTATGCTGGCACGTCGCTGTGAACGTCTGCTGGAA
CAGGCGTCTATGCTGGAACCGGGTTGGCTGCCGGACGCGCAGATGGAACTGCTGGACTTTTACCGTCGTCAGAT
GCTGGACCTGGCGTGTGGTAAACTGAGCCGTGAAGCTTaattaaggaggtaaaaaaaatgAAACGTGCGATCATCGTTGGCGGT
GGTCTGGCGGGTGGCCTGACCGCGATCTACCTGGCCAAACGTGGCTACGAAGTTCATGTTGTGGAAAAACGTGG
TGACCCGCTGCGTGACCTGAGCAGCTACGTTGACGTTGTTTCTAGCCGTGCGATCGGTGTTAGCATGACCGTTC
GTGGTATCAAATCCGTTCTGGCTGCGGGTATCCCGCGTGCGGAACTGGATGCTTGCGGTGAACCGATCGTTGCG
ATGGCGTTCAGCGTTGGTGGTCAGTATCGTATGCGTGAACTGAAACCGCTGGAAGATTTCCGTCCGCTGTCCTT
GAACCGTGCTGCGTTCCAGAAACTGCTGAACAAATACGCGAACCTGGCGGGCGTTCGTTACTACTTCGAACATA
AATGCCTGGACGTGGACCTGGATGGTAAAAGCGTGCTGATCCAGGGTAAAGATGGCCAGCCGCAGCGTCTGCAG
GGCGATATGATTATCGGTGCAGATGGTGCGCACTCTGCGGTGCGTCAGGCTATGCAGAGCGGCCTGCGTCGTTT
TGAATTTCAGCAGACCTTCTTCCGTCACGGTTATAAAACCCTGGTTCTGCCGGATGCGCAGGCGCTGGGCTACC
GTAAAGATACCCTGTATTTCTTCGGTATGGATTCCGGTGGCCTGTTCGCGGGCCGTGCGGCGACCATTCCGGAT
GGTTCTGTTTCTATCGCCGTGTGCCTGCCGTACAGCGGTTCTCCGTCCCTGACCACCACCGACGAACCGACCAT
GCGTGCGTTCTTTGATCGTTACTTTGGCGGTCTGCCGCGTGACGCGCGTGACGAAATGCTGCGTCAGTTCCTGG
CTAAACCGTCCAACGATCTGATTAACGTTCGTTCTAGCACCTTCCACTATAAAGGTAACGTTCTGCTGCTGGGTG
ATGCGGCGCACGCGACCGCCCCGTTCCTGGGCCAGGGTATGAACATGGCGCTGGAAGATGCGCGCACCTTTGT
GGAACTGCTGGATCGCCACCAGGGTGATCAGGATAAAGCCTTCCCGGAATTTACCGAACTGCGTAAAGTTCAGG
CGGATGCAATGCAGGATATGGCGCGTGCGAACTACGACGTTCTGAGCTGCTCTAACCCGATCTTCTTTATGCGC
GCGCGCTACACCCGTTACATGCACTCTAAATTTCCGGGCCTGTACCCGCCGGATATGGCGGAAAAACTGTACTT
CACCTCCGAACCGTACGATCGTCTGCAGCAGATCCAGCGTAAACAGAACGTTTGGTATAAAATCGGTCGTGTTA
ACACCTCTGGTGATTATAAAGATGATGATGATAAAtaattaaggaggtaaaaaaaatgGAAAACCGTGAACCGCCGCTGCTGCCG
GCTCGTTGGAGCAGCGCGTACGTTAGCTACTGGAGCCCGATGCTGCCGGATGATCAGCTGACCAGCGGTTACTG
CTGGTTCGATTACGAACGTGATATCTGCCGTATCGATGGTCTGTTCAACCCGTGGAGCGAACGTGATACCGGCT
ACCGTCTGTGGATGTCTGAAGTTGGCAACGCGGCGTCCGGCCGTACCTGGAAACAGAAAGTTGCGTACGGTCGT
GAACGCACCGCGCTGGGCGAACAGCTGTGCGAACGTCCGCTGGATGACGAAACCGGCCCGTTCGCGGAACTGT
TCCTGCCGCGTGATGTTCTGCGCCGTCTGGGCGCGCGTCACATTGGTCGTCGTGTGGTTCTGGGCCGTGAAGC
GGATGGTTGGCGTTACCAGCGTCCGGGCAAAGGTCCGAGCACCCTGTACCTGGATGCGGCGAGCGGTACTCCG
CTGCGTATGGTTACCGGCGATGAAGCGAGCCGTGCGTCCCTGCGTGATTTCCCGAACGTTAGCGAAGCGGAAAT
CCCGGATGCGGTTTTCGCGGCGAAACGTTAAAAGCTT

Note: The sequence in green shows metal-responsive regulators, the sequence with a turquoise background represents the constitutive promoter P*ceuR*, and the sequence with a gray background represents the divergent pbr or mer promoter. The Pb(II) sensing-responsive element inserted at the *Bgl*II–*Sac*I sites. The Hg(II) sensing-responsive element inserted at the *Sac*I–*Hin*dIII sites.

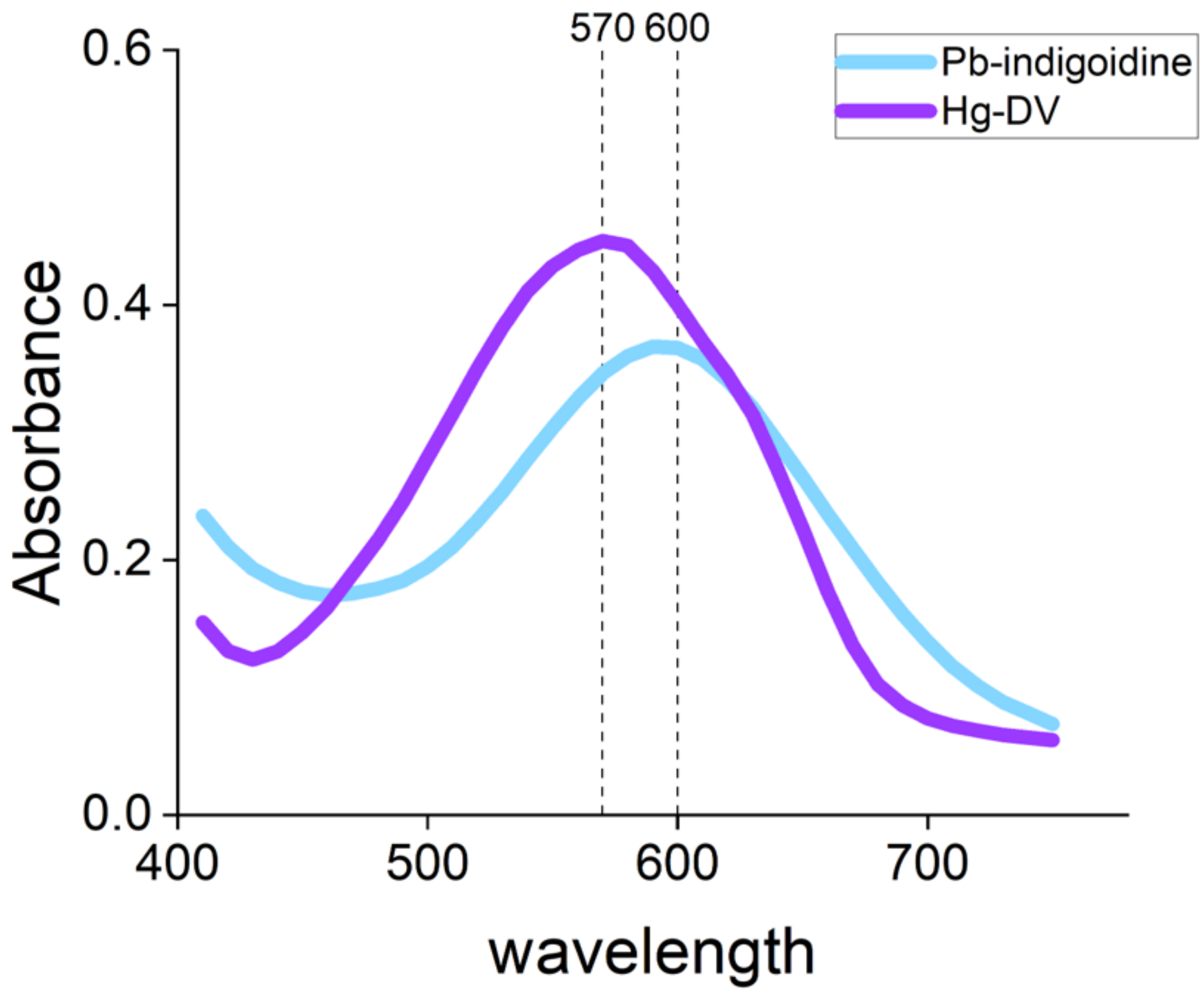

Fig. S1. Full-wavelength absorption spectra of the chromogenic products. The Pb-indigoidine product shows a maximum absorption peak at 600 nm, whereas the Hg-deoxyviolacein product shows a maximum absorption peak at 570 nm.

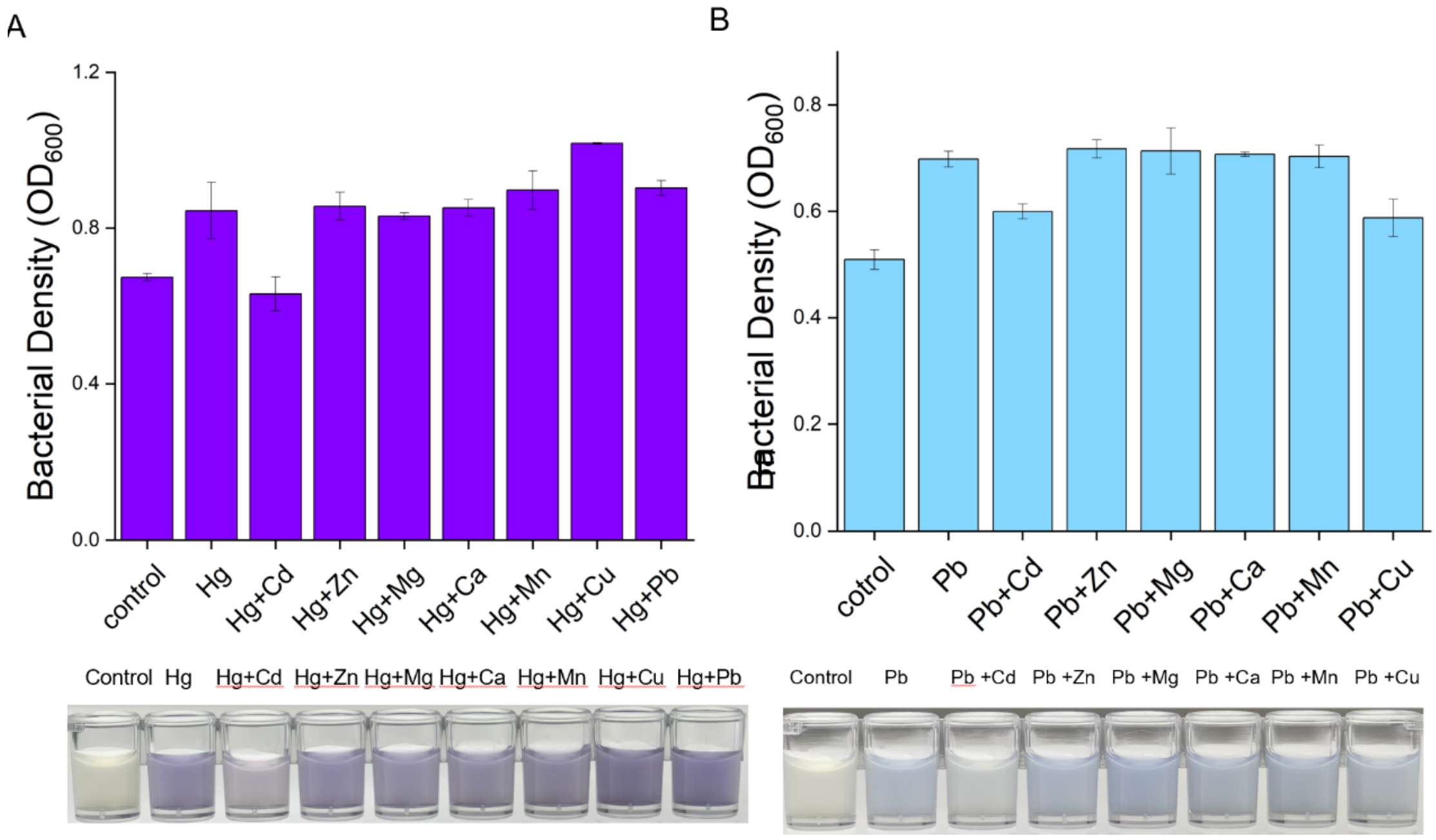

Fig. S2. Anti-interference performance of TOP10/pPb-IND-Hg-DV against co-existing

metal ions. The bar charts show bacterial density (OD₆₀₀), with representative photographs of the corresponding bacterial cultures shown below.

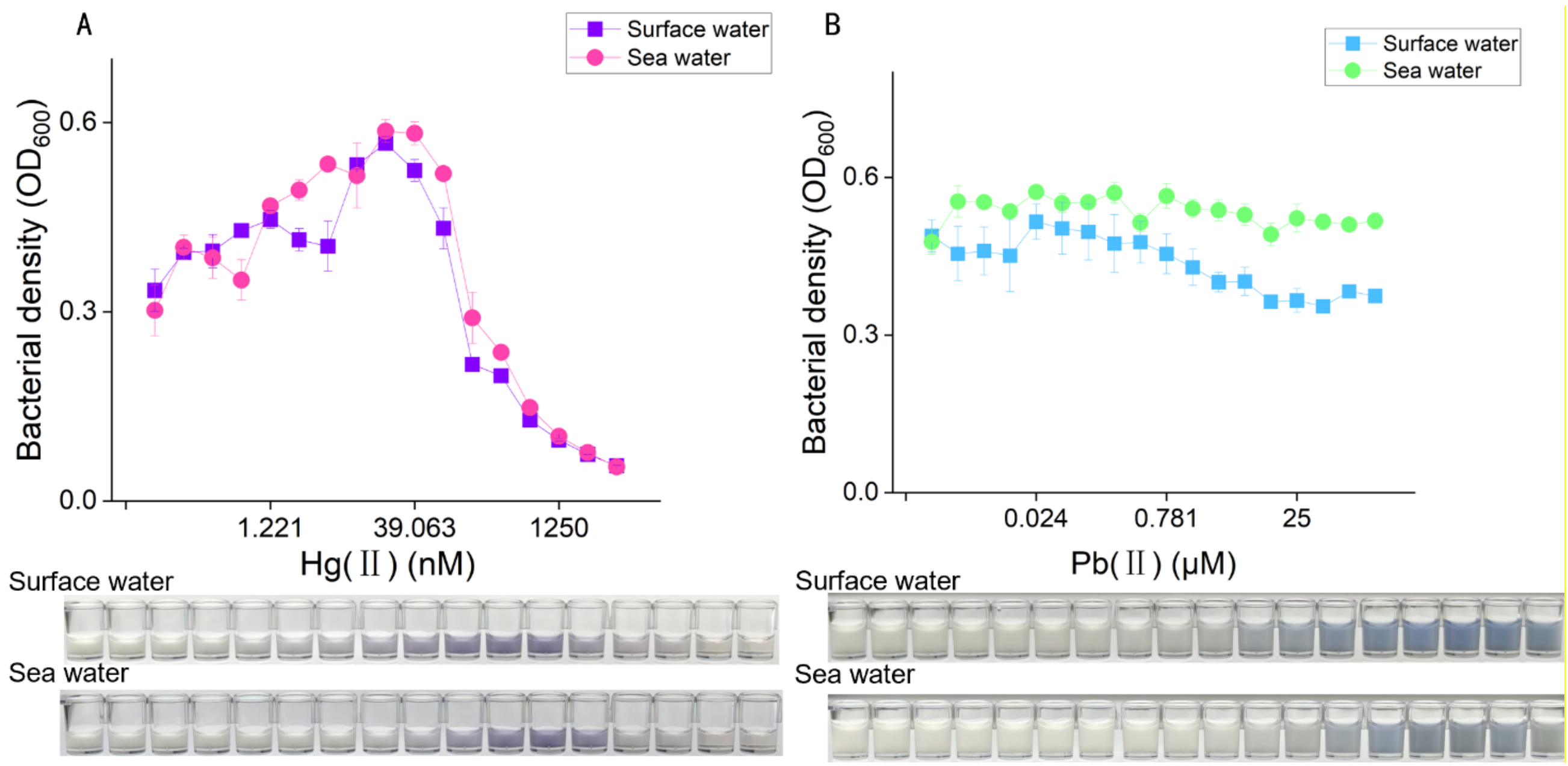

Fig. S3. Bacterial density (OD₆₀₀) of TOP10/pPb-IND-Hg-DV during exposure to Hg(II) (A) and Pb(II) (B) in surface water and seawater matrices.

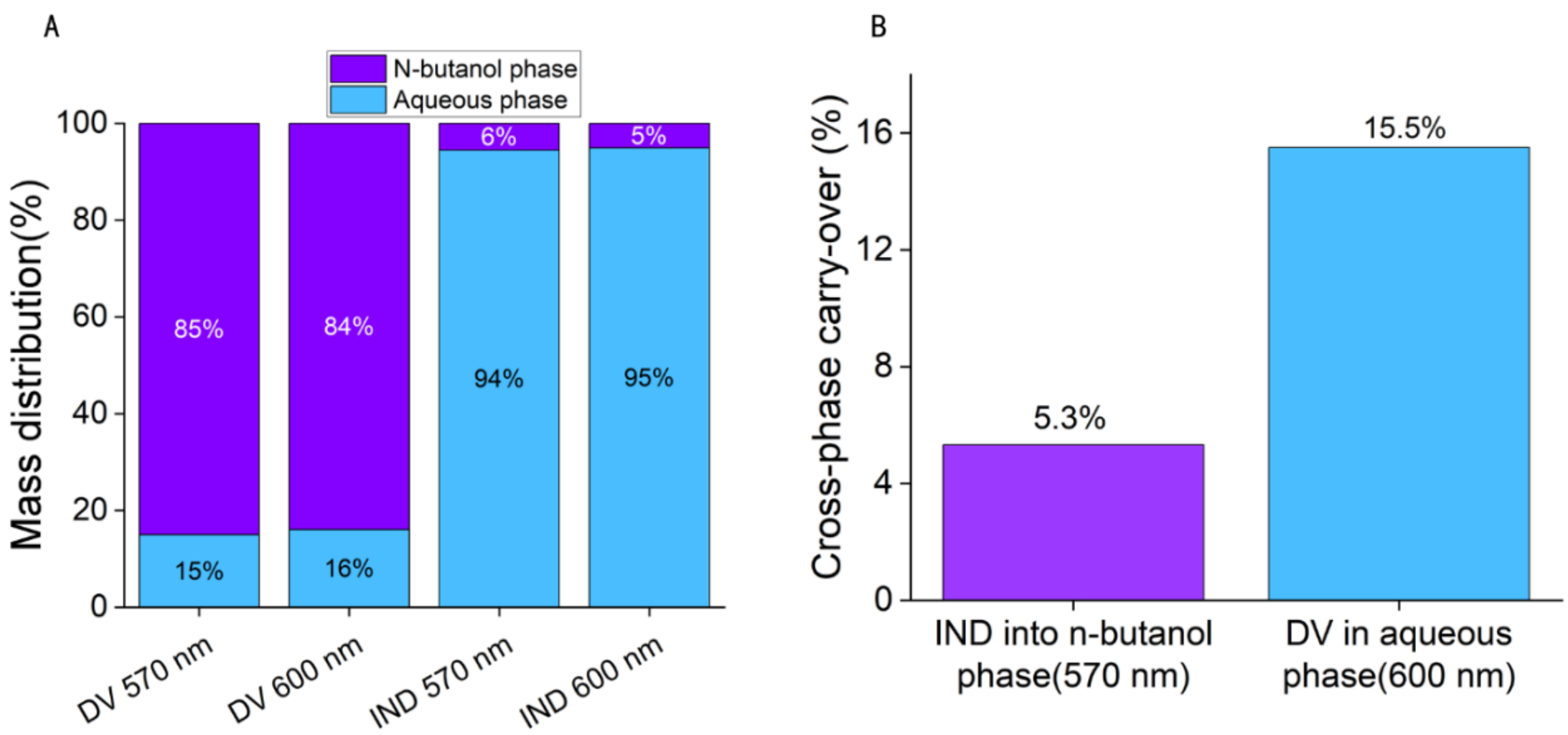

Fig. S4. (A) Volume-weighted distribution of independently prepared IND and DV between the aqueous (200 μL) and n-butanol (100 μL) phases, determined from absorbance at 570 and 600 nm. (B) Residual cross-phase carry-over into the opposite detection channel, i.e., DV contribution to the IND channel at 600 nm in the aqueous phase and IND

contribution to the DV channel at 570 nm in the n-butanol phase. Means of three independent replicates (n = 3).
